# Clinic-on-a-Needle Array toward Future Minimally Invasive Wearable Artificial Pancreas Applications

**DOI:** 10.1021/acsnano.1c03310

**Published:** 2021-06-22

**Authors:** Omri Heifler, Ella Borberg, Nimrod Harpak, Marina Zverzhinetsky, Vadim Krivitsky, Itay Gabriel, Victor Fourman, Dov Sherman, Fernando Patolsky

**Affiliations:** † Department of Materials Science and Engineering, the Iby and Aladar Fleischman Faculty of Engineering, Tel Aviv University, Tel Aviv 69978, Israel; ‡ School of Chemistry, Faculty of Exact Sciences, Tel Aviv University, Tel Aviv 69978, Israel; § School of Mechanical Engineering, the Iby and Aladar Fleischman Faculty of Engineering, Tel Aviv University, Tel Aviv 69978, Israel

**Keywords:** microartificial organs, silicon nanowires, field effect transistor, metabolites, nanosensors, microneedles, hydrogel

## Abstract

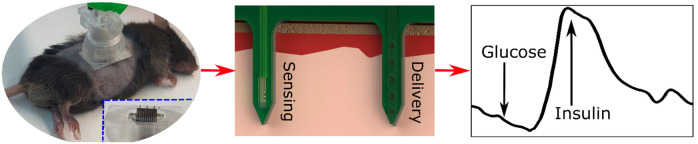

In order to reduce medical facility overload due to the rise of the elderly population, modern lifestyle diseases, or pandemics, the medical industry is currently developing point-of-care and home medical device systems. Diabetes is an incurable and lifetime disease, accountable for a significant mortality and socio-economic public health burden. Thus, tight glucose control in diabetic patients, which can prevent the onset of its late complications, is of enormous importance. Despite recent advances, the current best achievable management of glucose control is still inadequate, due to several key limitations in the system components, mainly related to the reliability of sensing components, both temporally and chemically, and the integration of sensing and delivery components in a single wearable platform, which is yet to be achieved. Thus, advanced closed-loop artificial pancreas systems able to modulate insulin delivery according to the measured sensor glucose levels, independently of patient supervision, represent a key requirement of development efforts. Here, we demonstrate a minimally invasive, transdermal, multiplex, and versatile continuous metabolites monitoring system in the subcutaneous interstitial fluid space based on a chemically modified SiNW-FET nanosensor array on microneedle elements. Using this technology, ISF-borne metabolites require no extraction and are measured directly and continuously by the nanosensors. Due to their chemical sensing mechanism, the nanosensor response is only influenced by the specific metabolite of interest, and no response is observed in the presence of potential exogenous and endogenous interferents known to seriously affect the response of current electrochemical glucose detection approaches. The 2D architecture of this platform, using a single SOI substrate as a top-down multipurpose material, resulted in a standard fabricated chip with 3D functionality. After proving the ability of the system to act as a selective multimetabolites sensor, we have implemented our platform to reach our main goal for *in vivo* continuous glucose monitoring of healthy human subjects. Furthermore, minor adjustments to the fabrication technique allow the on-chip integration of microinjection needle elements, which can ideally be used as a drug delivery system. Preliminary experiments on a mice animal model successfully demonstrated the single-chip capability to both monitor glucose levels as well as deliver insulin. By that, we hope to provide in the future a cost-effective and reliable wearable personalized clinical tool for patients and a strong tool for research, which will be able to perform direct monitoring of clinical biomarkers in the ISF as well as synchronized transdermal drug delivery by this single-chip multifunctional platform.

Diabetes is an incurable and a lifetime disease, accountable for a significant mortality and socio-economic public health burden. Also, the number of patients with diabetes is expected to highly and continuously increase in the future. Diabetes is a metabolic disorder classified into two main classes: type 1 and type 2 diabetes. Type 1 diabetes is a result of an autoimmune reaction that kills the β-cells in the pancreas, thus preventing the secretion of enough insulin. However, in type 2 diabetes, the individual develops a resistance to insulin accompanied by a significant β-cell dysfunction. Both cases result in an elevated concentration of blood glucose. Specifically, postprandial (i.e., after-meal) hyperglycemia is commonly defined as a blood glucose level greater than 180 mg/dL two hours after a meal.^1^ Chronic high blood glucose levels lead to pathological complications, including cardiovascular diseases, stroke, and potential nontraumatic limb amputations, as well as microvascular complications leading to kidney diseases, blindness, and neuropathy.^2,3^ Thus, tight glucose control in diabetic patients which can prevent, or at least delay, the onset of its late complications is of enormous importance and has been the subject of extensive research efforts over the years.^4^ Controlling blood sugar is also proven to prevent patient death resulting from loss of consciousness and heart failure during hypoglycemic events.^5,6^

Even though regulating blood glucose levels is a major concern for diabetic patients, satisfactory compliance with long-term testing is difficult to attain, given the discomfort of the repetitive nature of the commonly used finger-prick systems available commercially.^7−9^ Subcutaneously implanted systems able to monitor continuously glucose for up to 1 week, have then come to market to address the discomfort resulting from repeated injections. However, these systems require catheters that must be inserted in the subcutaneous tissue and replaced occasionally, and also potential chemical interferences hamper their application. Other approaches such as sweat and exhalation sampling, skin IR spectroscopy, and even contact lenses were investigated for metabolites monitoring, but they all lack the high blood correlation, metabolites variety, and sampling repeatability required for clinical applications.^10−12^ Furthermore, it has been shown that interstitial fluid (ISF) glucose concentrations parallel those of blood glucose concentration, and the same holds for additional metabolites such as lactate, amino acids, drugs, and more.^13−15^ In this context, the main challenge is the extraction of the extracellular fluid sample, since it is hard to separate this fluid from the surrounding cells in the tissue.^16,17^ In most research the ISF is extracted from the intradermal region by tempering the skin, which can cause inaccuracies.^18^

One of the most appealing concepts developed in recent years to monitor the ISF glucose levels is the microneedle-based systems. Due to their size, they have been demonstrated to be pain-free, minimally invasive, potentially low-cost, and easy-to-use platforms.^19^ Tissue was shown to recover completely 24 h after removal.^20^ Most microneedles developed nowadays are “vertical” needles rising from the surface in a 3D-architecture,^21−23^ aimed mostly and separately for glucose monitoring^24−28^ or drug delivery.^29−32^ While there are some advantages in this 3D-based architecture, the production steps required to fabricate microneedles are mostly unconventional and complicated, while the 3D drug delivery approach is typically passive.^26,30,33^ While some recent reports have shown various kinds of combinations involving glucose monitoring and drug delivery, these again are usually separately focused on the delivery system while the glucose level monitoring aspect is not analytically quantifiable.^34,35^

Silicon-nanowire field-effect transistors (SiNW-FETs) have shown great promise in regard to sensitively quantifying specific analytes even in physiological environments.^36−39^ SiNW-FETs hold multiple other advantages, including enhanced selectivity and stability in the presence of potential interferent molecules, in contrast to current amperometric (or coulometric) three-electrode electrochemical biosensors, which were vastly shown to be prone to detrimental analytical and clinical effects under the presence of a multitude of endogenous and exogenous chemical interferents.^40−42^ These interferent-related analytical artifacts are a result of the direct oxidation of the chemical interferents on the surface of the amperometric sensors at the working voltage conditions necessary for glucose detection. Although several approaches were investigated to overcome this limitation, most commercially available systems still suffer from chemical interferent-related issues, handicapping the future clinical applicability of these systems in artificial pancreas applications.

One of the most commonly used analytes for detection purposes is hydrogen peroxide (H_2_O_2_), which is produced by a variety of metabolic processes in the body and can be detected in biosamples by various techniques. In this regard, surface matching of a redox-active molecule, such as anthraquinone (AQ), can be used to detect metabolic activity due to its reactivity with reactive oxygen species (ROS) and H_2_O_2_. Modification of such a chemical moiety on the surface of the sensor can be used in SiNW-FET based systems for real-time, *ex vivo* selective detection of different metabolites.^43^ The selective redox reaction of the AQ chemical layer with hydrogen peroxide results in a significant change in the charge distribution of the participating surface molecules, thus leading to highly sensitive SiNW-FET sensing devices. Notably, by this NW-FET sensing scheme, unlike in current amperometric-based approaches, no direct electron transfer occurs between the nanowire sensing element and the detected metabolite (or hydrogen peroxide); thus, the presence of chemical interferents does not affect the analytical reliability of the sensing devices.

Furthermore, no system currently exists that utilizes all the advantages of analytical quantification and delivery via the integration of microneedles-embedded nanoFET devices and microneedle-based drug delivery elements integrated under a single on-chip platform.

In this work, a multiplex array of cointegrated sensing and delivery microneedle elements was created, based on a highly sensitive array of chemically modified redox-reversible SiNW-FET sensing devices capable of analytically monitoring *in vivo* and in real time several important small molecular metabolites, such as glucose and lactate, along with the simultaneous capability to deliver drugs through microfluidic-based needle elements on the same microchip platform. This integrated platform was clinically tested by the continuous monitoring of glucose levels in human volunteers, as well as in the mice animal model for the combined glucose monitoring and insulin delivery. The preliminary results obtained demonstrate the intrinsic capability of our platform to serve as a basis for the development of future wearable minimally invasive artificial pancreas applications.

## Results and Discussion

**Figure 1:**
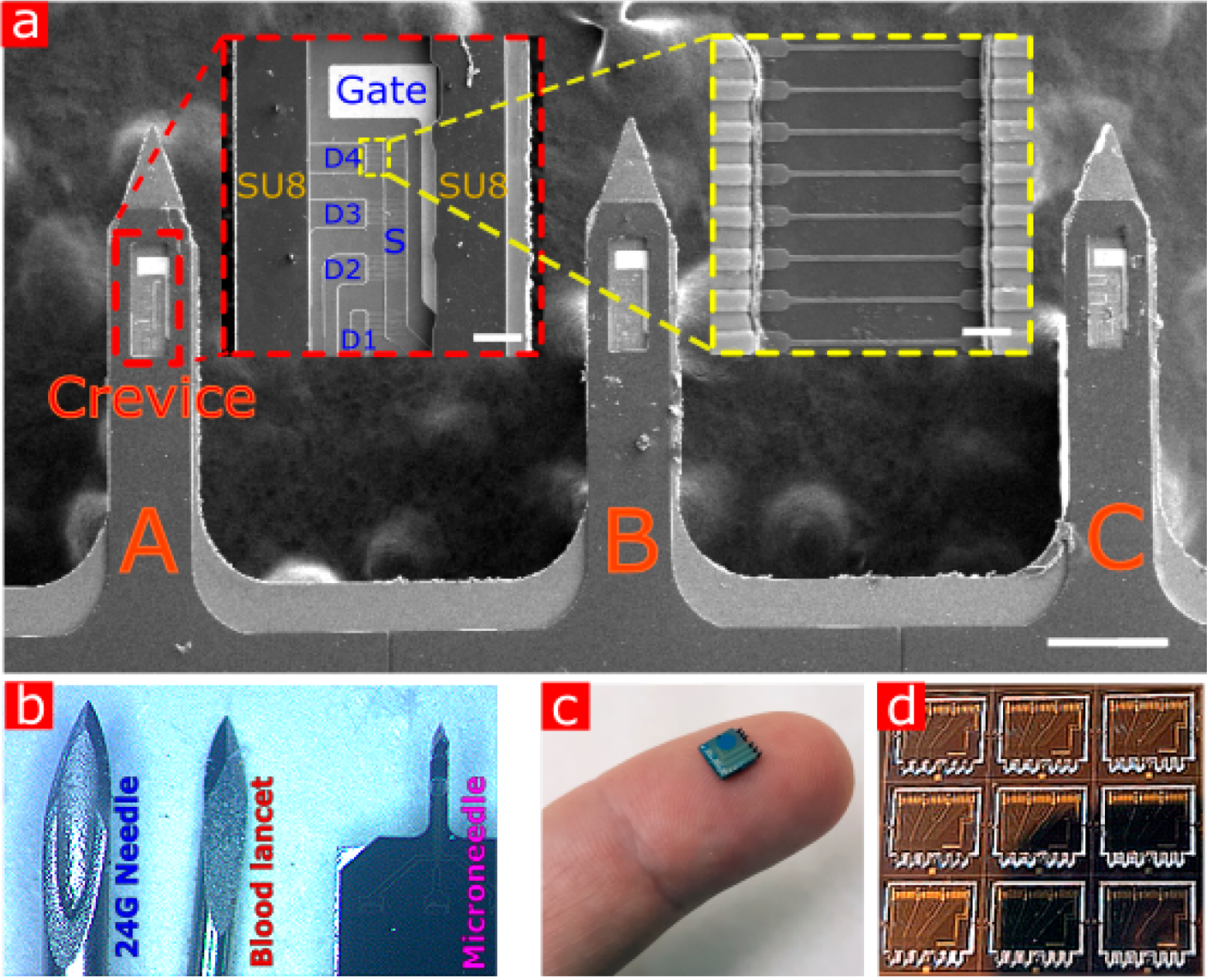
SiNW array-based metabolite sensor on microneedles concept. a, SEM image of the sensing chip layout. Fully fabricated self-limiting 700 μm long microneedles set, glued on carbon tape (scale bar: 250 μm). Red dashed inset shows the SU8 protected sensing crevice with four exposed SiNW-FET based devices (scale bar: 25 μm). Yellow dashed inset is the SiNW array (scale bar: 2 μm). b, Dimension comparison between our microneedle, commercially used injection needles, and the blood lancet. c, The microneedle chip on a finger. d, Die batch image of a six microneedle architecture after full separation by micromachining.

For the purpose of continuous transdermal monitoring, a vertical robust microscale silicon needle with an integrated electrical nanosensors array at its edge was developed. The principle of the platform, as shown in Figure 1a, is based on the following structure: (a) A depth self-limiting sharp silicon microneedle, (b) a polymer-based protection layer with a crevice exposing the sensing and gating regions, and (c) an independent multiple SiNW-FET sensor array on each needle. The red dashed inset in Figure 1a shows the sensing crevice protected by SU8, four SiNW-FET devices (D1–4), a gold gate, and passivated Ti/Pd source–drain contacts. The SiNW array device, with eight nanowires 125 nm wide and 50 nm high between contacts, is shown in the yellow dashed inset. The wider area of the SiNW is used to improve Si/metal contact. The use of three separate needles enables multiplex sensing, and the redundancy of the sensors in our platform allows for self-calibration by the use of enzyme-free microneedle elements; thus, a shift caused by a nonspecific reaction would be referred to as part of the baseline.

As can be seen in Figure 1b,c, the dimensions of the microneedles are significantly smaller than the commercially used needles and lancets for insulin injection and blood sampling; thus, our microneedles are minimally invasive and can be easily inserted compared to commercially applied needles and lancets.

Supporting Information Figure S1 depicts the fabrication process of the microneedles. The common micromachining techniques used to fabricate the multiplex sensing microneedles offer great architectural flexibility—the needles can be fabricated in different lengths to penetrate different desired tissues, and the nanowire-based sensing devices can be designed to fit any 2D topology of the needles. The microneedles are fabricated using a top-down approach,] and consist of only 2D fabrication techniques using standard micro/nanomachining methods, while resulting in 3D functional microneedles. The use of silicon-on-insulator (SOI) substrates allows for ease of the fabrication workflow, as no bottom-up techniques are required to complete the functional microneedles. This results in the ability to produce batch dies, as shown in Figure 1d, with highly controllable and repetitive electrical properties, which correlate to the number of wires that are fabricated and the SOI wafer properties.

**Figure 2:**
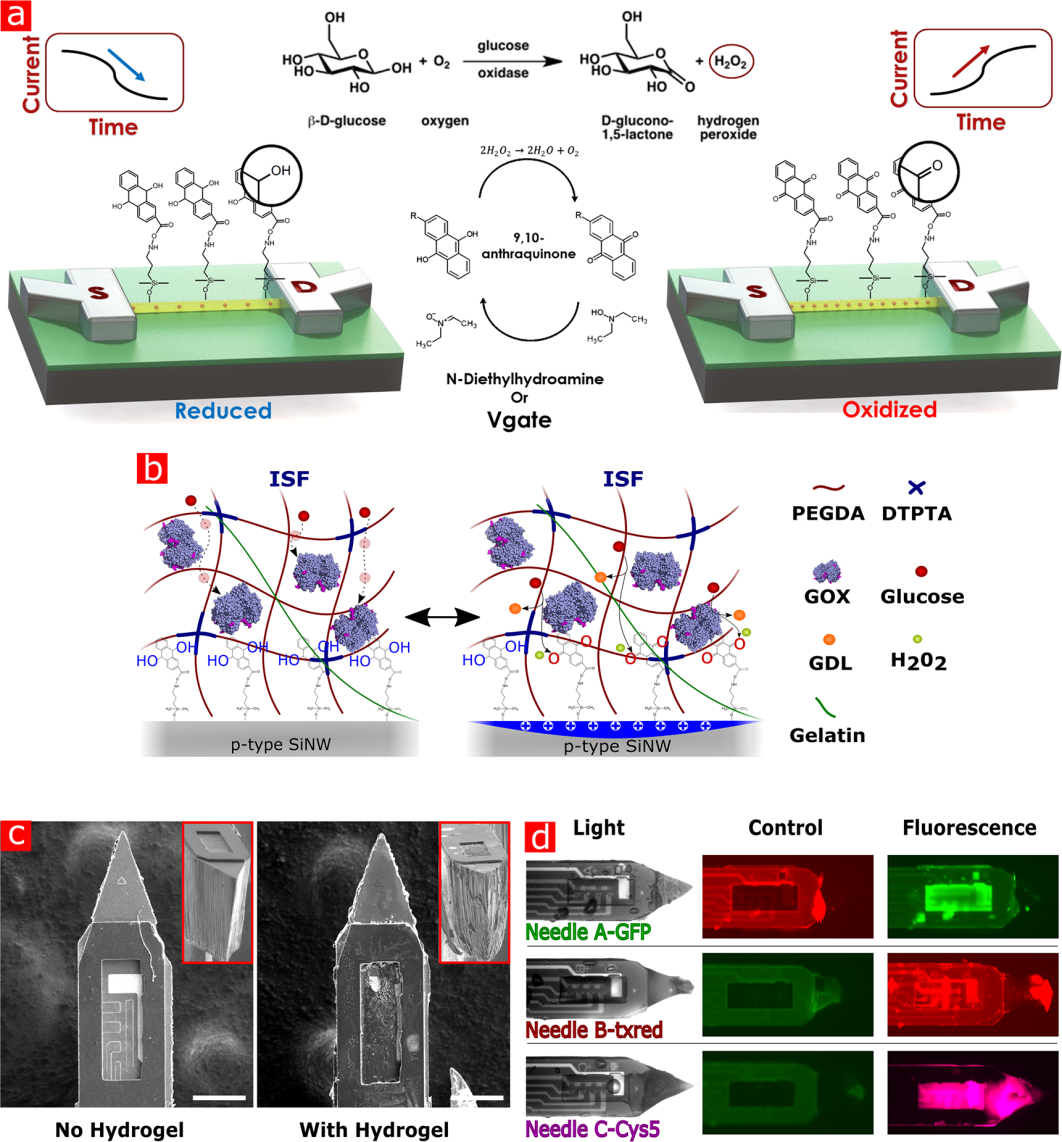
Principle of the chemically active SiNW-FET biosensor modified with AQ and hydrogel. a, Reaction diagram of AQ surface-modified SiNW-FET in the presence of metabolite and its oxidizing enzyme. b, Schematic of the hydrogel structure and glucose reaction mechanism applied on a SiNW device. c, SEM images of a Si microneedle with or without applied hydrogel (scale bar: 100 μm). The red contour inset shows a tilted image of the needles. d, Application of the hydrogel on microneedles set on the same die where different fluorescent species were added to the hydrogel of each microneedle. The control images are under excitation that does not induce emission of the specific fluorophore.

In order to enable transdermal monitoring of different metabolites, a chemical modification of the SiNW-FET surface is conducted to covalently bind an amino-silane derivative followed by covalent binding of the redox moiety AQ. Figure 2a shows the basic principle of metabolite sensing of our platform. The covalently bound AQ moieties on the SiNW surface specifically react with oxidizing species, such as H_2_O_2_, which leads to an alteration of the electric field of the SiNWs, resulting in a change of the conductivity. Consequently, the changes in measured currents of the FET can be directly linked to the concentration of the oxidizing species that react with the surface-bound AQ moieties. The fact that many oxidase enzymes, such as glucose and lactate oxidase (GOX and LOX, respectively), have H_2_O_2_ as a byproduct molecule in their chemical oxidation pathways allows exploiting the AQ surface modification for sensing the corresponding metabolites simply by utilizing these enzymes. The oxidation of the AQ molecule, when exposed to H_2_O_2_, is expected to increase the measured conductivity of the nanosensors, while an applied negative gate voltage that causes reduction of the enzymatically oxidized AQ moieties is expected to reduce the conductivity of the *p*-type FET SiNW devices.^44^ Here, for the application and immobilization of enzymes, a hydrogel embedding medium is used, while a SU8 protection area-confining layer is applied in order to create a crevice spanning the sensing SiNW-FET array area.

The surface modification process and X-ray photoelectron spectroscopy (XPS) characterization measurements are detailed in Supporting Information Figure S3. Modification of 3-aminopropyldimethylethoxysilane (APDMES) is carried out in the gas phase, under a vacuum, in order to ensure binding of an amino-silane monolayer. The redox-reactive anthraquinone moiety is then covalently attached to the amino-silane layer by using anthraquinone-2-carboxylic acid (AQCA) with a diisopropylcarboimide (DIC) linker in DMSO. XPS measurements, depicted in Supporting Information Figure S3b, provide an elemental composition comparison during the different steps of the surface modification. Prior to any modification process, the measured surface exhibits small amounts of carbon and nitrogen, which indicate mere contaminations. After APDMES modification, both the carbon and the nitrogen contents drastically rise. Surface binding of the quinone moieties results in a clear increase in the elemental carbon content, which may indicate good surface coverage and proper covalent binding of the quinone redox molecules to the sensing Si surface.

As schematically illustrated in Figure 2b, poly(ethylene glycol) diacrylate (PEGDA)-based hydrogel with a diethylenetriaminepentaacetic acid (DTPTA) linkers matrix is deposited over the area containing the SiNW-FETs sensing devices. This hydrogel layer encapsulates enzyme molecules, glucose, and lactate oxidase enzymes, immobilizing them in the hydrogel matrix while maintaining their catalytic activity and permittivity to glucose and lactate molecules. Furthermore, the upper side of the hydrogel layer is accessible to the ISF, allowing the diffusion of free glucose and lactate molecules into the gel layer for the final reaction with the specific embedded enzymes. The enzymes in the gel catalyze the oxidation of glucose, producing H_2_O_2_ (and glucono delta-lactone (GDL)) which diffuses toward the SiNW sensing device surface and oxidizes the AQ moieties, to an extent correlating the glucose concentration in the medium. To improve mechanical properties, by lowering stress-related effects,^45^ gelatin molecules were added to the hydrogel. Also, to lower the reaction rate and control other factors influencing the measurements (diffusion time, polymer swelling),^46,47^ the hydrogel thickness was limited to approximately 7 μm, by controlling the depth of the SU8 polymer crevice in the sensing area, as seen in Figure 2c. It is important to emphasize that implanting the needle elements into the skin tissue without negatively affecting the analytical performance of the sensor, by negatively affecting the active sensing devices or the covering enzyme-embedded hydrogel layer, is a critical factor when developing implantable devices. Sensing devices must be physically protected from the physiological environment during measurements and insertion. The physical configuration of our system allows this to be realized in preparation for *in vivo* experiments. While the basic AQ modification is identical on each needle, each crevice can be modified with a hydrogel layer modified with a variety of enzymes or active molecules. Figure 2d depicts different fluorescent dies that are mixed within the hydrogel matrix and applied to different individual needles. This needle-addressable specific chemical modification is the key to performing multimetabolite levels monitoring by a single-chip platform.

**Figure 3:**
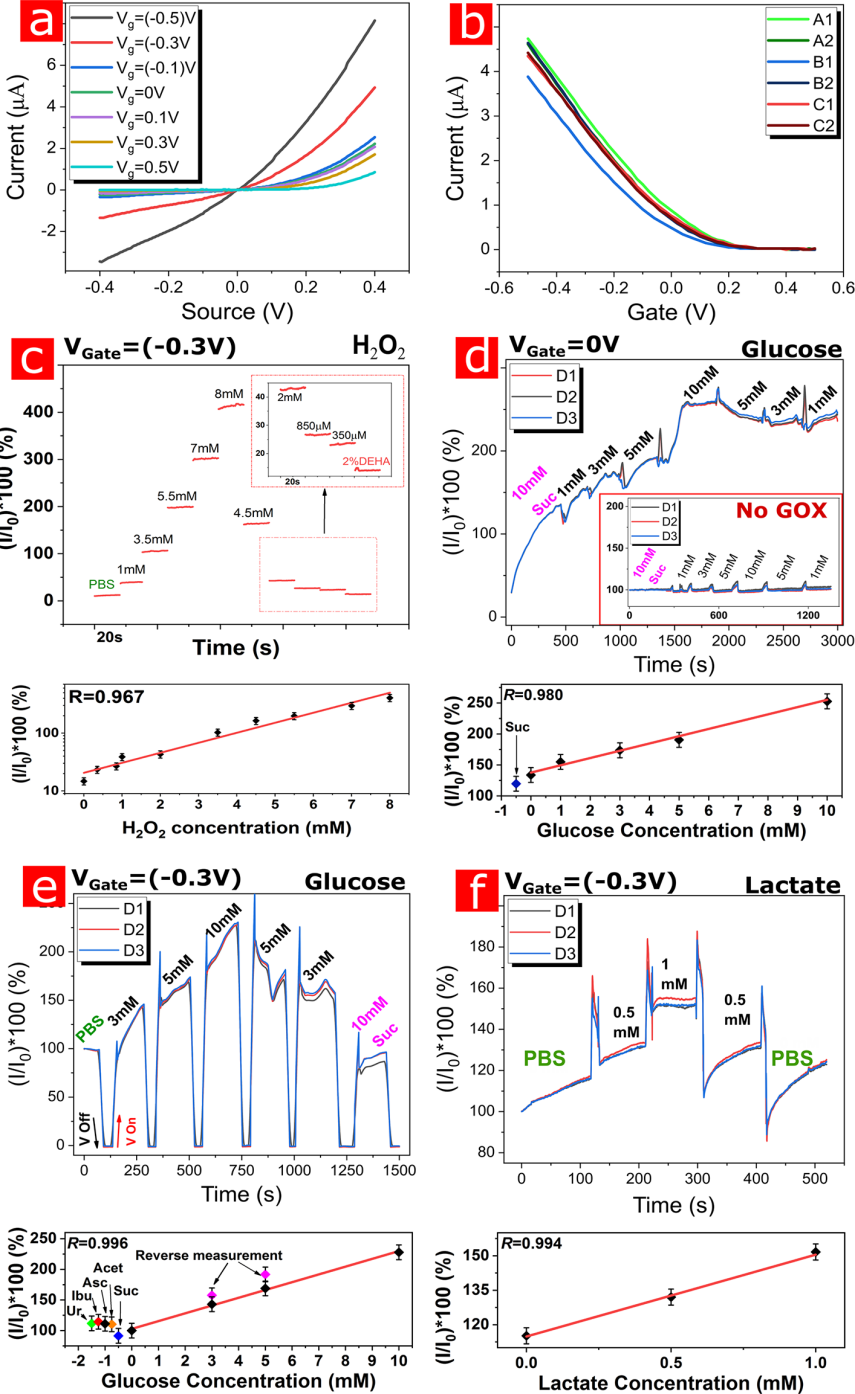
Electrical characterization and multimetabolites sensing. a, IV plot of a single device with a *V*_sd_ range of −0.4 to 0.4 V by a 0.1 V/s rate and *V*_gate_ intervals of 0.2 V. b, TC plot of two of the highest variance devices from each microneedle on a single die. The needles were immersed in DIW and *V*_sd_ = 0.2 V with a water gate ranging between −0.5 and 0.5 V by a 0.1 V/s rate and showing a typical *p*-type SiNW behavior. c, Reaction calibration measurement for H_2_O_2_ concentrations in PBS solution. d, Reaction calibration measurement for glucose concentrations in a gelatin medium without applying gate reduction, *V*_sd_ = 0.2 V. Inset: Measurements in the absence of GOX. e, Reaction calibration measurement for glucose concentrations in a gelatin medium with applied gat -reduction, *V*_g_ = −0.3 V, *V*_sd_ = 0.2 V. f, Reaction calibration measurement for lactate concentrations in a gelatin medium with applied gate reduction, *V*_g_ = −0.3 V, *V*_sd_ = 0.2 V.

The electrical characterization of the SiNW-FET device array is shown in Figure 3a,b. IV plots of a single device demonstrate an almost linear performance between *V*_sd_ −0.3 to 0.3 V, which is the region the devices are meant to operate in. It is vital that the device would operate in a *V*_sd_**–***V*_gate_ potential lower than 1 V to prevent water hydrolysis and maintain low power consumption performance. A TC (transconductance) plot of two of the highest variance devices from each microneedle on a single die showed a typical *p*-type SiNW behavior. The relative standard deviation under an applied gate of −0.3 V was calculated as 9.7%.

The hydrogel-embedded microneedle-based AQ-modified sensors display good sensitivity to H_2_O_2_, as shown in the calibration curve depicted in Figure 3c. The calibration curve was measured in 1× PBS solution (150 mM) at different concentrations of H_2_O_2_, showing that the hydrogel-embedded SiNW-FET nanosensor responses are not affected by the high salt concentration. The sensors are shown to be sensitive down to a concentration of 0.35 mM H_2_O_2_ and up to a concentration of above 10 mM. Notably, the basal concentration of H_2_O_2_ in human plasma and the ISF is known to be in the range of 1–5 μM, reaching up to a concentration of ∼50 μM under certain pathological conditions.^48,49^ This physiological concentration range for hydrogen peroxide is below the lowest limit of detection of H_2_O_2_ of our sensors. Therefore, these physiological μM levels of H_2_O_2_ should not interfere with the continuous measurement of glucose and other metabolites, with physiological concentrations in the higher range of mM. These H_2_O_2_ μM concentrations that may be potentially caused due to local tissue inflammation as a result of microneedle insertion are thus under the detection limit of our nanosensor devices. Additionally, our needles are inserted to a maximum depth of 600 μm, unlike current sensors for CGM that are inserted to the depth of ∼3–5 mm. The dimensions of our platform will cause fewer undesired physiological effects due to its considerably lower invasiveness.

The importance of the AQ moiety presence on the surface of the SiNWs nanosensors is presented in Supporting Information Figure S4. The APDMES-only modified nanosensors show sensitivity to the presence of hydrogen peroxide at considerably higher concentrations, Figure S4a, in comparison to the APDMES/AQ-commodified devices, Figure S4b. In addition, APDMES is not selective toward metabolites present in the ISF and can desorb from the surface during the first 24 h in aqueous solutions.^50^ Also, medical conditions such as acidosis and alkalosis are well-known physiological conditions that cause alterations in the blood, and therefore the ISF, pH levels. Thus, it is important to monitor the pH level, while sensing any metabolite *in vivo*, to extract the true signal of the analyte. Figure S4d depicts the sensitivity of the enzyme-free hydrogel-embedded microneedles-based sensors to the pH, showing that the microneedle-based sensor is sensitive to relevant physiological pH changes, which can help calibrate and differentiate the signal output arising from the detected analyte. Figure S4e shows the stability of the sensors in a PBS solution during continuous operation of 5 days, demonstrating the electrical stability of the resulting sensors under physiological conditions under continuous operation. It is important to note that beyond the ability to calibrate the sensors’ signals at different pHs, pH values in the ISF do not change beyond physiological values of 6.5–7.4. From the pH measurements using enzyme-free hydrogel embedded nanosensors, it can be seen that the change in current derived from these pH changes, pH in the range of 6.5–7.6, is only about 10%. Given that the changes shown in hydrogen peroxide/glucose measurements exceed 100% in the physiological range, such observed signal changes in this physiological pH range would not cause severe measurement errors in the monitoring of different metabolites. The result of such measurements can be seen in Supporting Information Figure S5.

**Figure 4:**
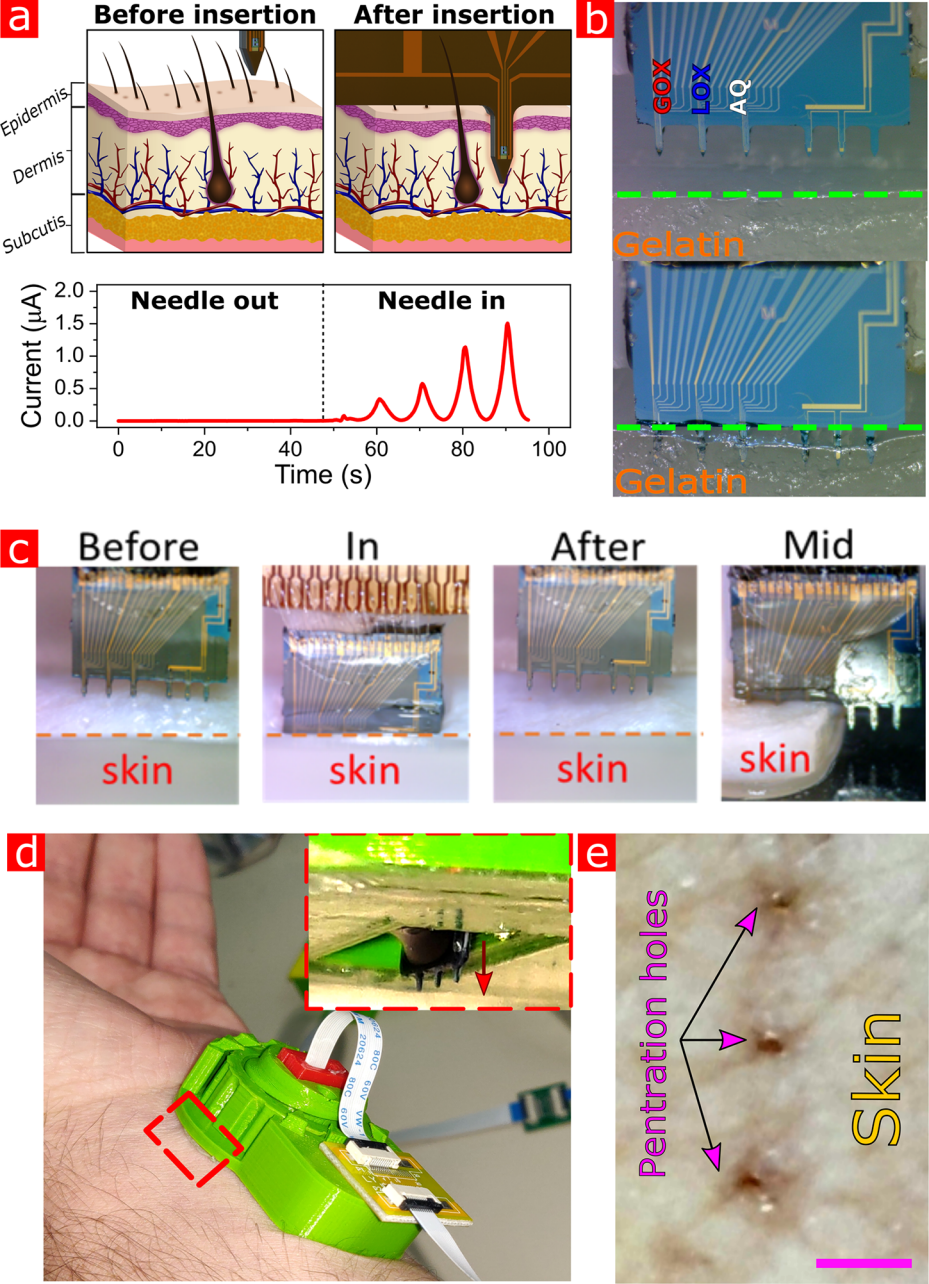
Microneedle platform ISF insertion. a, Illustration of microneedle penetration through the skin. Bottom: TC (*V*_gate_ = −0.5 to 0.5 V, *V*_sd_ = 0.2 V) while dry and in the ISF medium. b, Insertion of a multianalyte sensor, to a gelatin medium. The sensors are inserted into the gelatin by a 3-axis micromanipulator. The dashed green line marks the gel surface. c, Demonstration of 2 mm pig skin penetration and microneedle stability after insertion and extraction. The needles are penetrating the skin without effort and do not break during entry. Penetration is limited by the length of the microneedles (700 μm) and does not require force regulation. d, 3D printed patch, with a user-controlled dial for vertical microneedle insertion. The red dashed contour inset shows an inverted look on the microneedles passing through the patch apparatus. e, Penetration holes in the skin after the OGTT test (scale bar: 650 μm).

The surface oxidation of AQ moieties by H_2_O_2_ is a stable chemical process that results in a stable reoccurring electrical signal but unfortunately requires a chemical reduction step for reusability of the sensor. While a reducing agent can be used in some sensing configurations,^43,44^ mainly in *ex vivo* environments, *in vivo* continuous metabolic monitoring cannot allow the use of such reagents. Supporting Information Figure S6 depicts a simple 3 h long *ex vivo* experiment in which different H_2_O_2_ concentrations have been used for detection, and between each concentration, the microneedle-based FET was placed in a 2% DEHA reducing agent solution in order to completely reduce the AQ moieties. The DEHA reducing agent manages to completely reverse the oxidation of the AQ, caused by the presence of H_2_O_2_, returning the nanodevices to their original baseline currents after each chemical reduction step. During *in vivo* metabolites monitoring, such a reduction step can be alternatively achieved by a “hot electron” injection mechanism, directly achieved by changing the gate voltage of the nanodevices.^44^Supporting Information Figure S7 shows the effect of the AQ moiety reduction/oxidation by the application of a suitable gate voltage on XPS measurements performed on these devices. Upon reduction of the redox-sensitive AQ moieties by application of a gate voltage of −0.3 V, an increase in C–O counts can be observed, correlating to a successful double bond reduction in the quinone molecule (blue line). Conversely, the oxidized state, which exhibits a carbon–oxygen double bond, shows lower C–O counts (red and green lines).^51^ These experiments demonstrate the reversible nature of the surface-bound AQ moiety oxidation state while continuously monitoring different relevant metabolites under physiological *in vivo* environments. In order to simulate *in vivo* measurements, an *in vitro* gelatin-based skin-mimicking layer was applied over the testing solutions into which the multiplexed microneedle-based nanoFETs sensing array was vertically inserted, Figure 4b. The measurements were performed by using GOX- and LOX-embedded hydrogels deposited on individual needles on the same chip, while one microneedle was used for the self-calibration baseline, in order to extract the proper responses relating to the glucose and lactate levels, respectively. This skin-mimicking gelatin layer was placed over a reservoir of PBS solution containing varying concentrations of metabolites, to prevent gel dehydration through self-evaporation. The concentration of metabolites in the gelatin matrix was chosen according to hypo- to hyper-levels of glucose in human blood and ISF (3.5–10 mM for glucose, 0.5–1 mM lactate).^52,53^ To normalize the response of different devices, the SiNW-FET sensor signals are measured in a buffered medium in the absence of any metabolite, and the electrical responses are normalized according to eq 1.
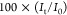
1where *I*_0_ is the current in a metabolite-free buffer and *I*_t_ is the current at a certain time point during the measurement in the presence of a metabolite at a certain concentration.

Measurements of different concentrations of glucose in the gelatin medium and the resulting calibration plots are shown in Figure 3d,e. Figure 3d shows the results of sampling skin-like gelatin layers spiked with different concentrations of glucose without applying the gate-reduction mechanism on the nanoFET devices. The sensors initially showed signals linearly correlated to increasing glucose concentrations, between 1 and 10 mM, and this is comparable to the range exhibited by commercial systems.^54^ The limit of detection was found to be 0.15 mM, with a detection range of 1–20 mM glucose. The sensors did not show signal reversibility when *V*_g_ = 0, and this shows the need for the application of a negative gate voltage in order to achieve a reversible sensing device, as suggested in Figure 2a.

Also, the control enzyme-free hydrogel-covered microneedle did not show any response to the glucose concentration change, thus demonstrating the high selectivity of our nanosensors array platform. However, Figure 3e shows that, after the application of a suitable gate voltage, reversible reduction of the AQ moieties is achieved, and the nanosensors display both linear and reversible responses to sequentially increasing and decreasing glucose concentrations. The concentration-dependent results clearly demonstrate stable and accurate glucose concentration readings, with minimal signal shift after hours of continuous operation. Additionally, Figure 3f shows the results of measurements performed by a LOX-embedded hydrogel-covered microneedle, with gate-voltage application, demonstrating a linear response to varying concentrations of lactate in the physiological range of 0–1 mM. The bottom plots represent the linear fit of detection values and *R* values of points taken after signal stabilization. Standard error values between devices are presented but unnoticeable, thus demonstrating the low variability in the nanosensors’ response. Notably, GOX-embedded nanosensors in “microneedle 1” do not react to the addition of lactate in the physiological range, as well as LOX-embedded “microneedle 2” do not react upon addition of glucose in the tested range. As said, the enzyme-free microneedle 3 element does not react to any of the added metabolites, thus demonstrating the capability to perform multimetabolite specific monitoring using our multiplexed platform. It is noteworthy to mention that current, amperometric or coulometric, commercially available glucose monitoring systems use a three-electrode setup configuration to perform direct redox reactions between the sensor metal surface and the biofluid tested metabolites (or their byproducts as H_2_O_2_), resulting in direct electron oxidation/reduction of these molecules on the surface of the electrode. These sensors are known, proved through multiple laboratory and clinical studies, to suffer from chemical interference (due to overpotentials applied), causing various endogenous or exogenous molecules to undergo direct oxidation reactions as well. Molecules such as acetaminophen, ascorbic acid, ibuprofen, urea, and various sugars, including mannitol, have all been proved to cause detrimental interferences to proper glucose measurements and so affecting their clinical deployment in future artificial pancreas applications.^41,42^ In contrast, as shown in the bottom panel of Figure 3e, our sensors, due to the selective redox reaction between H_2_O_2_ and the the AQ moiety and the absence of direct electron transfer between glucose (or the potential interferents) and the nanowire elements, do not allow these interferent chemical species to oxidize on the nanowire surface. Therefore, the proposed sensors show high selectivity against the metabolites of interest and interfering molecules do not elicit any sensor’s response. As shown in this figure, chemical interferents such as 0.1 mM acetaminophen, 2 mM iboprufen, 0.2 mM ascorbic acid, 0.5 mM uric acid, and 20 mM mannitol do not elicit any signal changes at their relevant clinical concentration range. Importantly, the intrinsic redundant nature (multiplexity) of our platform allows for accurate measurements, which may help to reduce potential error during real-time, *in vivo* measurements, as opposed to current technologies which are based on the use of a single sensing electrode, potentially leading to measuring errors and variances between devices.^55^

The importance of the applied gate voltage on the oxidation state of the surface-bound AQ molecules and the signals received during metabolites sensing is apparent in the comparison between Figure 3d and Figure 3e. In both experiments, the selectivity of the devices is demonstrated by the addition of high concentrations of sucrose (>10 mM) which do not result in any change in the sensors’ responses. In the control GOx-free hydrogel embedded microneedle, Figure 3d inset, no response to added glucose is detected since no H_2_O_2_ is produced in the absence of the enzyme GOx. When no gate voltage is applied, the signal of the GOx-embedded nanosensors rises with increasing concentrations of glucose, as more H_2_O_2_ is produced, resulting in the oxidation of increasing amounts of AQ moieties on the SiNWs surface. When the chip is reintroduced to lower levels of glucose, however, the signal does not return to its original amplitude, as the AQ molecules remain in their oxidized state irreversibly. However, when a negative gate voltage of −0.3 V is applied to the nanosensors, Figure 3e, the sensors are capable of monitoring fluctuating concentrations of metabolites reversibly, as the ampero-FET devices keep the oxidized-versus-reduced population of the AQ moieties at equilibrium. This applied negative gate voltage effectively and reversely reduces the AQ molecules and allows continuous glucose monitoring. Lactate monitoring was also established, with an LOX-hydrogel with added lactate, and shows reversible sensing capabilities for lactate as well, Figure 3f. All results show a linear signal increase, correlating nicely with the measured metabolite concentration. While in this study only glucose and lactate were studied, it is possible to monitor additional metabolites of clinical relevance, such as beta-butyrate in cases of ketosis, by the combination of the enzymes beta-butyrate dehydrogenase and NADH oxidase that produces hydrogen peroxide as a result of their enzymatic cascade.^56,57^

As could be seen in Figure 3e, the glucose response is stable after multiple insertion and extraction events into an ISF-mimicking gelatin medium, indicating that the concentration-dependent electronic signal is not affected by penetration events. Thus, the analytical capabilities of our platform are unharmed due to device skin insertion, owing to the geometrical configuration of the nanosensor-embedded needle, where the active devices are inside a 3D crevice on the needle and protected by a hydrogel layer.

Figure 4a schematically illustrates the insertion of the microneedle elements into the skin layers and the expected resulting TC measurements, respectively. When the needle has not yet penetrated the skin, no changes in the devices’ currents are noticed. Once the needles penetrate the epidermis into the dermis, the hydrogel embedded FET nanodevices are exposed to the ISF medium which, in turn, brings the clear appearance of TC curves.

An example of an experiment being performed on a skin-like gelatin medium with the multianalyte microneedles platform, where each of the three sensing microneedles (left needles) is covered by hydrogel with different enzymes, is shown in Figure 4b. The sensors are inserted into the gelatin by a 3-axis micromanipulator. In preparation for *in vivo* experiments, the microneedles’ mechanical tolerance applying an *ex vivo* pig skin model was examined to ensure skin penetration and stress resilience. Figure 4c shows the vertical insertion of the microneedle system to a 2 mm pig skin sample, which closely imitates the human skin. The needles exhibit a smooth entry, without breaking during entry for hundreds of penetration trials. It is noteworthy to mention that the penetration depth is limited by the length of the needles. To receive quantifiable results of the force needed for the microneedles to penetrate the skin, a mechanical test was conducted to measure the load required for penetration through stretched pigskin. Supporting Information Figure S8a shows the experimental setup for penetration load measurements. After insertion, penetration holes can be observed on the pig skin sample, as shown in Supporting Information Figure S8b, yet no residues were visible. To simulate different skin behaviors, the pigskin was penetrated both in a free-standing model as well as a PDMS-supported mode (for a stiffer insertion platform). Each experiment was performed four times, exhibiting similar results. With no support, the penetration to the skin required a force of approximately 1 N, while the stiffer setup required approximately 0.2 N of force for the needles to penetrate. The needles did not break even with 5 N of applied pressure. The results are comparable to previous microneedle penetration results.^58,59^ For the purpose of *in vivo* monitoring of metabolites, the forearm was selected as the microneedles insertion point due to the thinner epidermis layer in this body area. A custom-made wearable patch, as shown in Figure 4d, was 3D printed to accommodate the microneedle chip and allow its simple vertical insertion. The resulting penetration holes found in the skin immediately after insertion are shown in Figure 4e. Importantly, no scaring was observed following microneedle insertion to the forearm; see Supporting Information Figure S9. The minimally invasive microneedle architecture results in significantly reduced pain and discomfort levels as compared to finger-prick or commercial devices, due to its 600 μm depth restriction. As compared to commercially available needles, the pain has been reported to be drastically reduced or eradicated, giving the suggested microneedle-based sensor great advantage in terms of lack of discomfort during long, continuous monitoring.^60^

**Figure 5:**
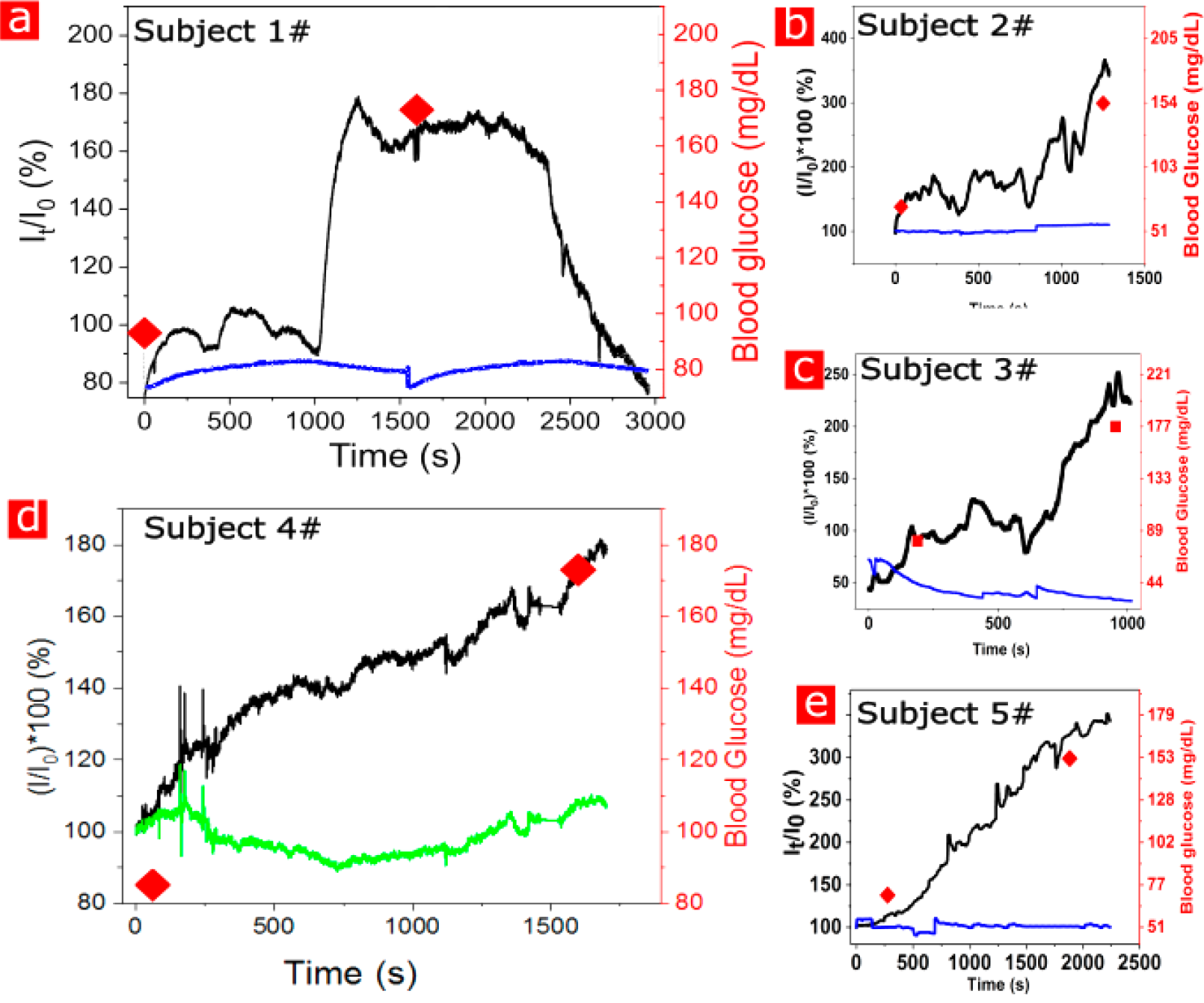
*In vivo* continuous glucose monitoring of the oral glucose tolerance test. *In vivo* glucose level measurements after 12 h of fasting (*V*_gate_ = −0.3 V, *V*_sd_ = 0.3 V). The 75 g oral glucose tolerance testing (OGTT) is monitored by a microneedle modified with a GOX-embedded hydrogel, black curve. Baseline measurements taken on an unmodified microneedle, blue curve. Red dots represent the measured glucometer blood glucose values. Lactate is monitored by a microneedle modified with an LOX-embedded hydrogel, green curve. Healthy subjects showed a sharp incline 20–25 minutes after glucose consumption. a–e correlate to subjects 1–5, respectively.

To measure the glucose response, we have conducted oral glucose tolerance tests on several human subjects (OGTT) after 12 h of fasting. Before, during, and after the test, the blood glucose concentration was tested using a commercial glucose meter (Accu-Chek) for comparison, as depicted in Figure 5. Once the sensor has penetrated the skin and been allowed to stabilize, a 2–3 minute period, the subject consumed 75 g of glucose in a period of 5 minutes and remained stationary for the rest of the examination. Five human volunteers were tested and showed a similar temporal response to glucose intake. While each individual person is expected to display a unique response curve, the timing and profiles had overlapping characteristics. The Subject #1 examination, Figure 5a, displayed a long-term stable signal before glucose consumption, followed by a signal increase as only measured by the GOx-embedded microneedle sensors, well correlated to the glucose measurements by the commercial glucometer, and after 25 minutes of continuous measurement, the signal reached its peak stabilization, followed by a signal decline corresponding to a decrease in the concentration of glucose in the ISF (as measured by the commercial glucometer). The observed signal pattern was repetitive to all subjects tested. These results correlate well with several studies showing a similar temporal response.^61,62^ All experiments shown in Figure 5 show a response to glucose consumption (black curves), correlating with blood glucose levels (red dots),^63^ while other devices on the same chip modified with Lox enzyme or control needles covered with enzyme-free hydrogel show a stable temporal electrical baseline and did not react to the ISF increase glucose concentrations resulting from the oral glucose consumption (blue curve). These results demonstrate the stability and high selectivity of our proposed platform under *in vivo* operation conditions. Figure 5d depicts a multiplexed measurement where one microneedle was modified with a GOx-embedded hydrogel and another microneedle with a LOx-embedded hydrogel. The LOx-embedded nanodevices show no significant reaction to increasing glucose concentrations, while the GOx-embedded nanodevices clearly and analytically respond to the applied glucose challenge step. Furthermore, the slight increase in lactate shown in the measurement after a period of 20 minutes following glucose consumption is correlative to past studies of lactate concentrations upon glucose administration after fasting.^64^Supporting Information Figure S10a shows the similarities of different devices across different individual microneedles. The variance in current, once the electrical signals have been normalized, is less than 10%, and the overall behavior across all nanodevices is shown to be the same. This shows the important aspect of redundancy, which may in the future allow for more accurate glucose monitoring measurements. Both *in vivo* and *in vitro* experiments have shown less than 13% signal variance between devices for a given glucose concentration (in relation to a standard glucose solution), also exhibiting great stability, accuracy, and sensitivity across individual nanodevices. Notably, no microneedle-based systems had yet demonstrated the capability of performing *in vivo* selective multimetabolite monitoring, not affected by the presence of potential molecular interferent species. It is noteworthy to mention that none of the materials used here provides any harmful issue regarding the biocompatibility of the microneedle-based devices. Past investigations have shown that both silicon and PEGDA-based hydrogels (including the cross-linker) are biocompatible, showing that the hydrogel exhibits a negligible degradation, in an *in vivo* environment, for over 10 weeks (due to continuous hydrolysis of the ester end groups).^65−71^

**Figure 6:**
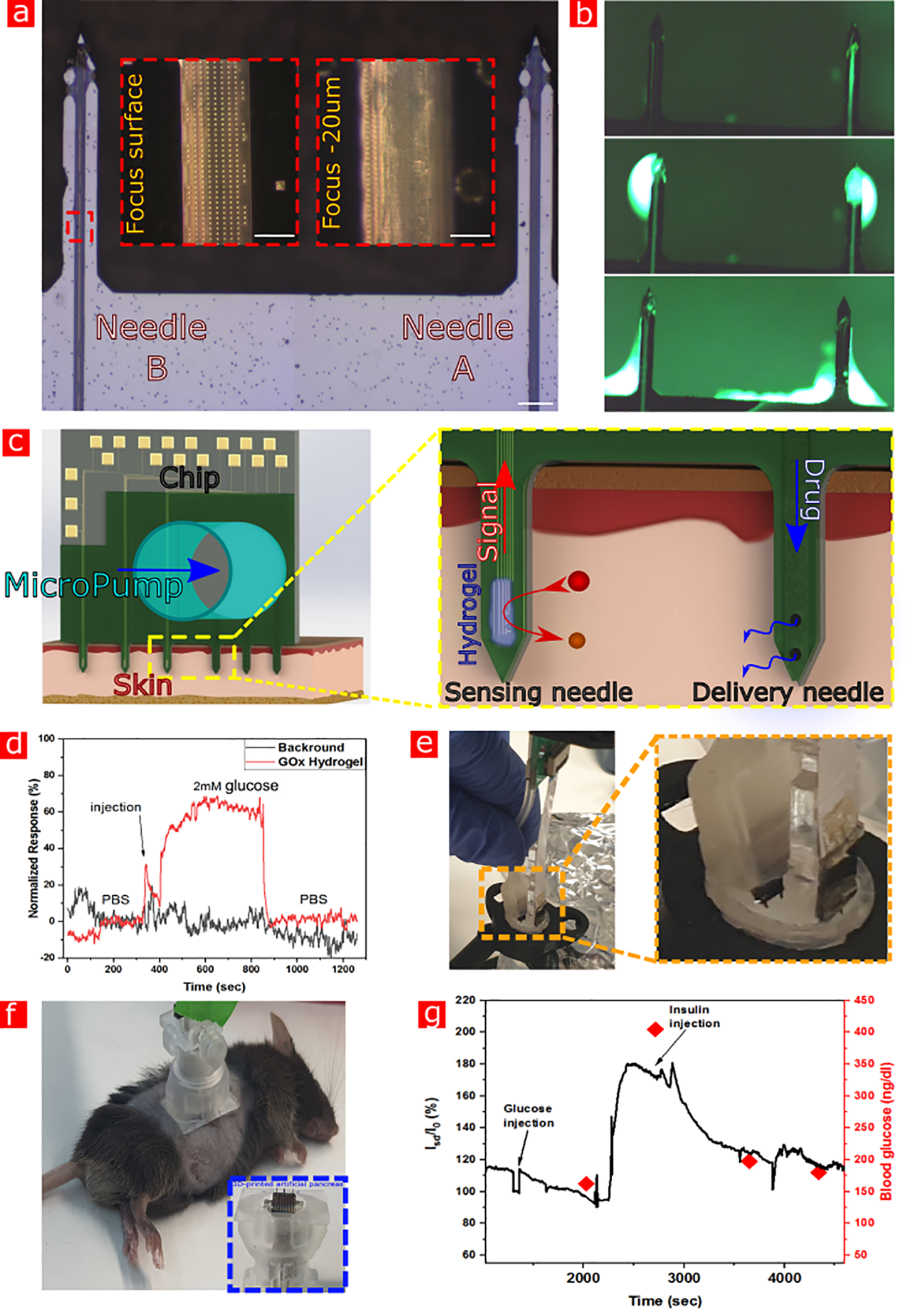
Microneedles with an embedded microfluidic system for drug delivery. a, Microscope image of two microneedles with a 5 mm long microchannel (scale bar: 20 μm). Red dashed insets show the array of cavities forming the channel (scale bar: 20 μm). b, Images of fluorescent solution ejection through two separate microfluidic microneedles. The top caption was taken just before the fluid release from the edge of the microneedle. At the bottom, the solution is withdrawn by the microneedles. c, Illustration of the clinic-on-a-needle platform. The integrated chip, connected to a bundled electrical monitoring system and micropump/reservoir device, penetrates the skin and is immobilized. The sensing microneedle hydrogel reacts with the metabolites of interest, sending a signal to the controller which initiate drug release until the sensor feedback signals to cease injection (yellow contour inset). d, *In vitro* simultaneous sensing and injection experiment results. Glucose injected by our on-chip microneedle element. e, Both delivery microneedle and sensor microneedle elements in a tube during the experiment. f. Sedated C57bl6 mouse with a 3D-printed artificial pancreas patch holding the microneedles chip in place during a continuous glucose measurement. Blue dashed contour inset shows an inverted look on the microneedles passing through the patch apparatus. g, *In vivo* mouse glucose level measurements after 16 h of fasting (*V*_gate_ = −0.3 V, *V*_sd_ = 0.3 V). The 2 g/kg GTT monitored by a microneedle sensor array modified with a GOX-embedded hydrogel, black curve. Insulin injection arrow depicts the time when human recombinant insulin was injected to the mice through the on-chip microneedle delivery element. Red dots represent blood glucose values measured using a commercial glucometer.

Additionally, as a preliminary proof of concept, a silicon-based microneedle drug delivery component was developed, as shown in Figure 6a. A top-down method for producing microfluidic channels (as depicted in Supporting Information Figure S11) is essential for the integration of a single multipurpose sensing + delivery chip. The microchannel width, length, and depth were controlled by the design of an array of 500 × 700 nm cavities in the passivation layer over the Si substrate (Figure 6a, inset), and the plane determined the etching rate of Si by tetramethylammonium hydroxide. As observed in Figure 6b under a fluorescent microscope, the solution fills the tunnels and is released only at the edge of the microneedles when positive pressure is applied. In order to show the capabilities of these microneedles to inject aqueous solutions, the needles were placed in a 3D-printed syringe with a fluorescent solution injected through the channels, at a measured rate of 54 μL–60 μL per minute. Importantly, the injection rate could be controlled by the force applied in the range of 10 nL–60 μL per minute. The diameter of the delivery channels is limited only by the width of the microneedle and can be designed to have an injection rate similar to needles with a gauge 34G, which is commercially used for insulin administration. It is noteworthy to mention that the fluid withdrawal action can be performed as well by applying negative pressure, making these delivery microneedles a tool for extremely low volume sampling applications. The integration of sensing and delivery microneedle elements in a single-chip platform allows the creation of a feedback-loop system able to simultaneously monitor glucose concentration and regulate glucose blood levels through insulin delivery in a similar manner to a human pancreas, thus operating as an artificial pancreas with minimally invasive properties, as schematically displayed in Figure 6c.

Demonstrating the mechanism of simultaneous injection and sensing, we performed *in vitro* sensing measurements using a microneedle chip, while injecting glucose via the on-chip microneedle delivery element to the tube, Figure 6d,f. Importantly, background measurements (black curve) show no response to the injection of glucose.

Notably, preliminary mice animal-model measurements have clearly shown the capability to perform both mentioned tasks simultaneously and effectively by our on-chip integrated platform, Figure 6f,g. Furthermore, direct injection of insulin near the glucose-measuring site does not interfere with the continuous measurements.^72,73^

For mass-scale production and clinical applications, interdevice variability needs to be lowered by achieving highly reproducible fabrication procedures to prevent variabilities in the nanodevices’ electric response. Also, future artificial pancreas applications require effective and reliable algorithms in order to control insulin delivery as a function of the measured glucose levels and are a key component in a complete integrated platform, deployable for clinical use.

## Conclusions

We have demonstrated a minimally invasive, transdermal, multiplex, and versatile continuous metabolite monitoring system in the ISF based on the SiNW-FETs nanosensors array embedded on microneedle elements. Using this technology, ISF-borne metabolites require no extraction and are measured directly by an adjustable hydrogel matrix with an oxidase enzyme. The 2D architecture, using a single SOI substrate as a top-down multipurpose material, resulted in a standardly fabricated chip with 3D functionality. Using a mimicking gelatin-based medium and *ex vivo* mammalian skin model provided us the insights for evaluating the sensor reaction to metabolites, potential chemical interferents, and mechanical forces applied on the microneedles and guided us for the right design of the supporting systems. After proving the ability of the system to act as a multimetabolites sensor, we have successfully applied our platform to reach the main goal for *in vivo* CGM of healthy human subjects. We were also able to develop an additional aspect of our system, allowing drug release through microinjection needle elements. The microneedle-nanosensors elements and the injection microneedle elements can be fabricated on the same chip with minor process adjustments. Preliminary animal-model experiments have shown the basic capability to perform both tasks by a single-chip platform.

Unlike other wearable approaches for glucose monitoring, the multiple sensing microneedles ensure the ability for multimetabolite sensing with increased accuracy during *in vivo* continuous monitoring. By that, we hope to provide a cost-effective and reliable wearable personalized clinical tool for patients and a strong tool for research, which will be able to perform direct monitoring of clinical biomarkers experiment in the ISF as well as synchronized transdermal drug delivery by this single-chip multifunctional platform.

## Experimental Section

### Substrates, Enzymes, and Reagents

The following chemicals were supplied by Sigma-Aldrich (now MERCK): phosphate buffered saline (PBS), *N*,*N*′-diisopropylcarbodiimide (DIC), 1-hydroxybenzotriazole (HOBt), Anthraquinone-2-carboxylic acid (AQCA), d-glucose (GLU), PEG-diacrylate Mn575 (PEGDA), pentaerythritol tetraacrylate, diphenyl(2,4,6-trimethylbenzoyl)phosphine oxide (DTPO), agar (A1296 powder), tetrakis(dimethylamido) hafnium(IV), glucose oxidase (GOX; G2133), *N*,*N*-diethylhydroxylamine (DEHA), sodium l-lactate (LAC), and tetramethylammonium hydroxide (TMAH). Dimethyl sulfoxide (DMSO), hydrogen peroxide (H_2_O_2_), and sulfuric acid were supplied by Biolab. *N*-Methyl-2-pyrrolidone (NMP), acetone, and 2-isopropanol (IPA) were supplied by J.T Baker. LOR5A, SF15, PMMA A4, and MMA el6 were supplied by Microchem (now Kayaku Advanced Materials). Lactate oxidase was from A.G Scientific. Hexamethyldisilazane (HMDS), AZ1505, and AZ4562 were supplied by Microchemicals. (3-Aminopropyl)-dimethyl-ethoxysilane was supplied by Gelest. SOI and Si wafers were supplied by SOITEC and University Wafers (device layer 50 nm 10 Ω per cm, BOX 150 nm, handle 725 μm, 10 Ω per cm, both handle and device layers were ⟨0-0-1⟩).

### Substrate Preparation

The 30 × 30 mm^2^ squares were first cleaned using acetone, isopropanol (IPA), and deionized water (DIW), followed by piranha solution (1:3 H_2_O_2_/sulfuric acid) and oxygen plasma for 2 minutes at 50 W. These substrates were subsequently thinned in order to provide easy penetration into the skin. The dry-chemical etch was performed by a Plasma-Therm VERSALINE DSE III bosch process over a photoresist pattern. To improve photoresist adhesion to the silicon surface, the substrate was treated with a hexamethyldisilazane (HMDS, Microchem) vapor prime process under 110 °C. The substrate backside was coated with 15 μm of an AZ 4562 (Microchem) photoresist layer by double-spinning the resist at 2000 rpm and baking it at 115 °C for 1 minute after each spin. The patterning etch area was made by mask-aligner photolithography (MA/BA6, Suss). The substrate was then aligned in a MA6 system followed by exposure of 360 mJ/cm^2^ in 60 mJ intervals and 25-s pauses. The exposed substrate was then immersed in water for 15 minutes to allow N_2_ gas dispersion, followed by development in an AZ 400 K (Microchem) 1:4 solution for 4 minutes. Finally, the substrate was postexposure baked for 10 minutes on a 120 °C hot plate and treated by oxygen plasma before DSE. The 550 μm DSE process was achieved in ∼750 cycles. After the DSE process, the residue of the resist layer was removed in a hot *N*-methyl-2-pyrrolidone (NMP) solution and hot piranha-solution treatment.

### Silicon Nanowire Synthesis

Silicon nanowire synthesis is preformed after patterning using e-beam lithography. The marker patterning is done by photolithography over the device layer by using the MA6 mask aligner backside alignment option. The resist layer consists of Lor5A resist (4000 rpm, 180 °C for 5 minutes) and AZ 1505-layer photoresist (4000 rpm, 110 °C for 1.5 minutes). The pattern is created by exposure of 14 mJ/cm^2^, developed in the AZ726 developer for 1 minute and rinsed with water. After the substrate is cleaned in oxygen plasma, a layer of 5 nm Cr and 30 nm Au is thermally deposited on top of the device layer. The process is completed by immersing the substrate in a hot NMP bath for the resist and excess metal lift-off. The substrate is coated by a 300 nm MMA EL6 and PMMA A4 (600 nm combined), both spin-coated at 5000 rpm and prebaked on a 180 °C hot plate for 3 minutes. The pattern is generated by exposing the resist at 130 μC/cm^2^ at 10 kV. After the exposure, the sample is developed in 1:3 MIBK/IPA for 1 minutes and cleaned in oxygen plasma. The masking is completed by coating 5 nm Cr and 30 nm Au in an electron-beam evaporator, followed by subsequent lift-off in acetone. Wire etch is then performed by dipping the sample in 10% HF for 5 s, followed by 10% TMAH solution treatment at 65 °C while stirring the solutions, for 30 s. The mask is removed by immersing the sample in gold etchant for 1 minute followed by chromium etchant for 2 minutes and then rinsing thoroughly with DIW.

### Fabrication of the Silicon Nanowire Field-Effect Transistor Array

Gate electrodes and bonding contacts of 5 nm Cr and 60 nm Au are then thermally evaporated over a photolithography patterning of Lor5A and AZ1505 resist in the same manner described earlier. The sample is treated in hot NMP for lift-off and in 5 minutes of UV/ozone at 65 °C. The contacts for the SiNW are patterned the same way as the Au layer in the previous stage and comprise 5 nm Ti, 100 nm Pd, and 30 nm Ti deposited by an electron-beam evaporator. Preceding evaporation, the sample is treated by 5 min of oxygen UV plasma and 1:6 BOE for 10 s. Passivation of the sensor contacts is carried out by forming a 120 nm silicon oxynitride in low-temperature PECVD and 5 nm alumina in the ALD process. After the evaporation, the sample is treated in hot NMP for lift-off and in 5 minutes of ozone UV followed by RTP of 450 °C for 20 s. In some samples, SU8 is used as a mechanical protection of the dies and especially of the sensing area of the needles. The sensing area, which is 40 μm wide and 80 μm long, is exposed for sensing. The SU8 layer consists of two different SU8 photoresist series. The first layer, which functions as an adhesion layer for the next thicker layer, is made by spin-coating SU8 2000.5 at 2000 rpm and prebaking it for 5 minutes over a 95 °C hot plate. The layer is patterned by 60 mJ/cm^2^ UV dose exposure. The sample is then treated by a postexposure bake on a 95 °C hot plate for 1 minute followed by a 1 minute development in SU8 developer solution. The second layer is made by spin-coating SU8 3005 at 1300 rpm and prebaking the sample on a 65 °C hot plate for 1 minute, and then the temperature is turned up to 95 °C for 10 minutes. The layer is patterned by exposure to 150 mJ/cm^2^. The sample is then treated by a postexposure bake on a 65 °C hot plate for 1 minute which is then turned up to 95 °C for 4 minutes. The SU8 resist is developed for 5 minutes in the SU8 developer, rinsed in isopropanol, and treated in ozone UV for 5 minutes. Finally, the SU8 layer is hot-baked for 20 minutes on a 180° hot plate.

### Needle Sensor Die Micromachining and Separation

The separation of dies by micromachining is done by a DSE process similar to that used for the backside. The Lor10B layer was spin-coated at 2500 rpm and prebaked at 170 °C on a hot plate for 7 minutes. A single 8 μm-thick layer of photoresist is enough to achieve the separation and was created by spin-coating the photoresist at 2500 rpm and prebaking at 115 °C on a hot plate for 1 minute. The etching pattern was generated by exposure of 250 mJ/cm^2^ in a mask aligner at 50 mJ intervals and 25 s pauses. The development of the resist was the same as described before. Since the top side of the SOI substrate has an oxide layer of 150 nm with an etching selectivity of up to 1:200, the SiO_2_ layer is first etched in an Oerlikon RIE system by CF_4_ gas at 250 W for 5 minutes. The sample is then processed in the Versaline DSE system for 270 cycles. Once this is done, the dies are separated from the batch-sample frame and cleaned by DDW, rinsed in a stream of acetone for 30 s, and immersed in RT NMP for 1 h. The dies are then characterized electrically, treated by ozone UV for 5 minutes, and immersed in ethanol for 30 minutes.

### Anthraquinone Surface Modification

The surfaces of mounted chips are then chemically modified. First, samples are treated by ozone UV for 5 minutes. Then, they are modified with organo-amino-silane APDMES in a vacuum oven for 4 h at 100 °C. Once the oven reaches vacuum, the pump is disconnected from the chamber. After the process is complete, the samples are rinsed in a stream of IPA for 1 minute, dried in N_2_, and baked at 70 °C for 10 minutes, covered by a Pyrex plate.

After the amine modification, the samples are modified with anthraquinone. The modification is achieved by the use of anthraquinone-2-carboxylic acid (AQCA) and a carbodiimide for the amide-bond reaction. Since the AQCA does not dissolve in aqueous solutions, the reaction was carried out in DMSO solvent. A solution of 50 mM *N*,*N*′-diisopropylcarbodiimide (DIC, Sigma), 50 mM 1-hydroxybenzotriazole (HOBt), and 10 mM AQCA was prepared, and the chips were modified by incubating the mounted chips for 2 h at room temperature, while carefully immersing only the needle region in the solution. After that time period, the chips were rinsed in DMSO, isopropanol, PBS, DDW, and isopropanol again and dried in N_2_.

### Hydrogel Matrix Surface Modification

The hydrogel solution is prepared by mixing 65% PEGDA Mn575, 3% pentaerythritol tetraacrylate, 2% gelatin from porcine skin (∼300 g Bloom), and glucose oxidase in PBS. Because the PEGDA solution contains 400–600 ppm 4-methoxyphenol (MEHQ) as the inhibitor, the PEGDA and pentaerythritol tetraacrylate were mixed and transferred through two inhibitor-remover columns and by centrifuge. Once all the components mixed, the solution was divided into 200 μL Eppendorf tubes. Just before manually covering the hydrogel over the needles, 5 μL of 30 mM diphenyl(2,4,6-trimethylbenzoyl)phosphine oxide (DTPO) in ethanol was added to the hydrogel mixture. The hydrogel was vigorously mixed and gently applied over the chip’s needles by a human hair and cleaned by sonication in 70% ethanol for 5 minutes. Excess hydrogel is wiped with parafilm. The sensing area of the needles is surrounded by SU8 walls, which create a crevice for the hydrogel, and encapsulated by hydrogel. Finally, the hydrogel polymerization is achieved by 385 nm 9 mW UV-lamp exposure for 10 s under a stream of N_2_ and incubation overnight at 2 °C–8 °C in a humidified dish.

### *In Vivo* Studies Using Mice

The *in vivo* performance of our integrated sensing + delivery platform was tested on adult healthy mice (male C57BL/6 mice; 6–8 weeks of age). The animal protocols were approved by the Tel Aviv University Committee on Animal Care. One mouse was fasted for 16 h and received an intraperitoneal injection of 2 g/kg glucose, followed by human recombinant insulin 30 minutes later (insulin dose: 1 U/kg). The blood glucose levels were monitored every 15 minutes following injection (glucose or insulin) with a commercial Accu-check Performa blood glucose meter and monitored continuously using our microneedle system. Blood glucose was measured from tail vein blood samples (∼3 μL).

## Supporting Information Available

Detailed experimental section, schematic illustration of the microneedle-based FET fabrication, images of batch fabrications, schematic illustration and XPS measurements of the chemical modification process, pH sensing and stability of the microneedle-based FET, comparison to H_2_O_2_ sensitivity with and without AQ, experimental results of H_2_O_2_ sensing in a spiked solution of PBS and 10% serum, XPS results of gate-induced AQ, images of microneedle insertion in pigskin, variance between different individual needles during *in vivo* experiments, and schematic illustration of fluidic channel fabrication (PDF)

## Notes

Approval for the *in vivo* human experiments was received from the Ethics Committee of Tel Aviv University. Animal *in vivo* experiments were approved by the Tel-Aviv University Animal Care Committee.
