# Supplementary material for: Clinic-on-a-Needle Array toward Future Minimally Invasive Wearable Artificial Pancreas Applications
Source: ACS Nano. 2021 Jun 22;15(7):12019–33. doi: 10.1021/acsnano.1c03310 (PMC8397432; doi:10.1021/acsnano.1c03310)
Supplement: Supplementary file 1 — nn1c03310_si_001.pdf [file nn1c03310_si_001.pdf]

Supporting Information

Clinic-On-A-Needle Array Towards Future Minimally-Invasive Artificial Pancreas Applications

Omri Heifler[1], Ella Borberg[2], Nimrod Harpak[2], Marina Zverzhinetsky[2], Vadim Krivitsky[2], Itay Gabriel[1], Victor Fourman[3], Dov Sherman[1,3] and Fernando Patolsky[1,2*]

1. Department of Materials Science and Engineering, the Iby and Aladar Fleischman Faculty of Engineering, Tel Aviv University, Tel Aviv 69978, Israel.
2. School of Chemistry, Faculty of Exact Sciences, Tel Aviv University, Tel Aviv, 69978, Israel.
3. School of Mechanical Engineering, the Iby and Aladar Fleischman Faculty of Engineering, Tel Aviv University, Tel Aviv 69978, Israel.

Email: fernando@post.tau.ac.il.

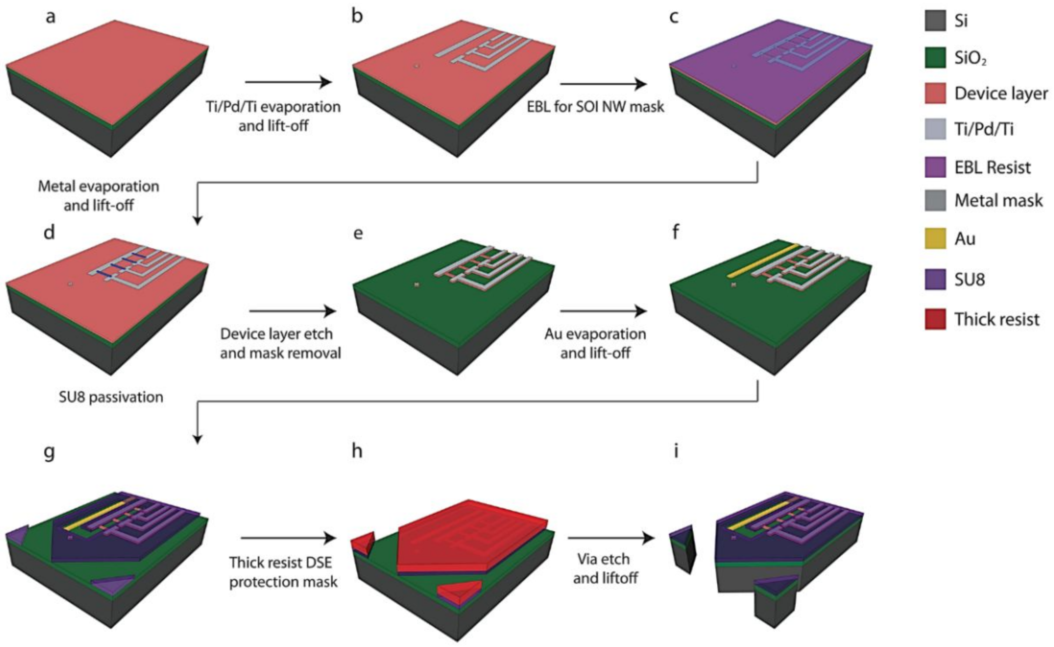

Figure S1| Microneedle sample fabrication steps:

a) SOI substrate is cleaned; b) Metal contacts evaporated; c) EBL process open NW windows; d) Metal mask evaporation and lifoff; e) TMAH etch and mask removal; f) Au pads and gate evaporated; g) SU8 passivation; h) Thick resist mask; i) DSE micromachining and mask liftoff;

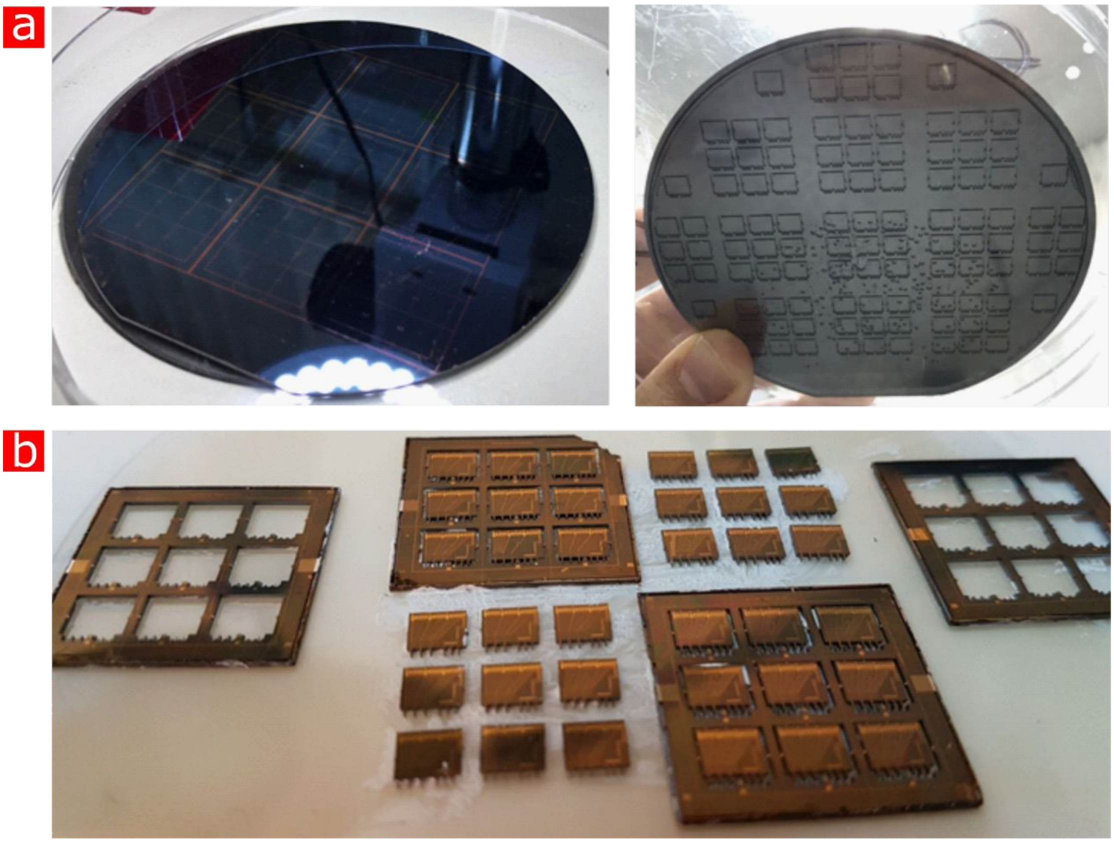

Figure S2 | 4-inch SOI substrate microneedles in batch dies:

Demonstration of fabrication in a larger scale. **a,** On the left, the top side of the substrate, revealing the metal contacts and sensors. On the right, the bottom side with halfway deep etch, for microneedle thinning and reducing top etch time. **b,** The dies after etching process. Some of the frames were removed for display.

a

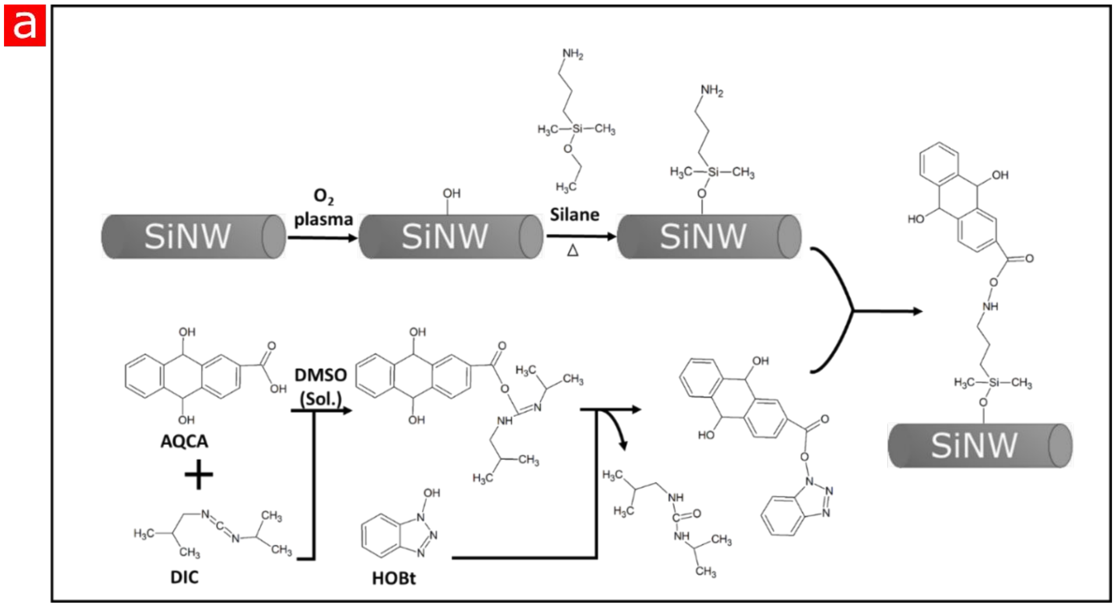

b

	Plasma treated Si/SiO_2 substrate	APDMES	APDMES+AQ+ DMSO	AQ+DIC +DMSO without APDMES	APDMES+AQ+DIC +DMSO
C	2.51	18.19	28.08	21.29	44.72
O	64.82	49.01	44.26	50.45	30.17
Si	32.14	29.42	24.64	26.88	20.61
N	0.14	3.27	2.88	1.38	3.72

Figure S3 | AQ modification surface modification steps and XPS verification:

a, Schematic illustration of the amino-silane and anthraquinone modification process. **b,** Comparison between 5 Si/SiO_2 samples, with different chemical treatments, XPS surface measurement.

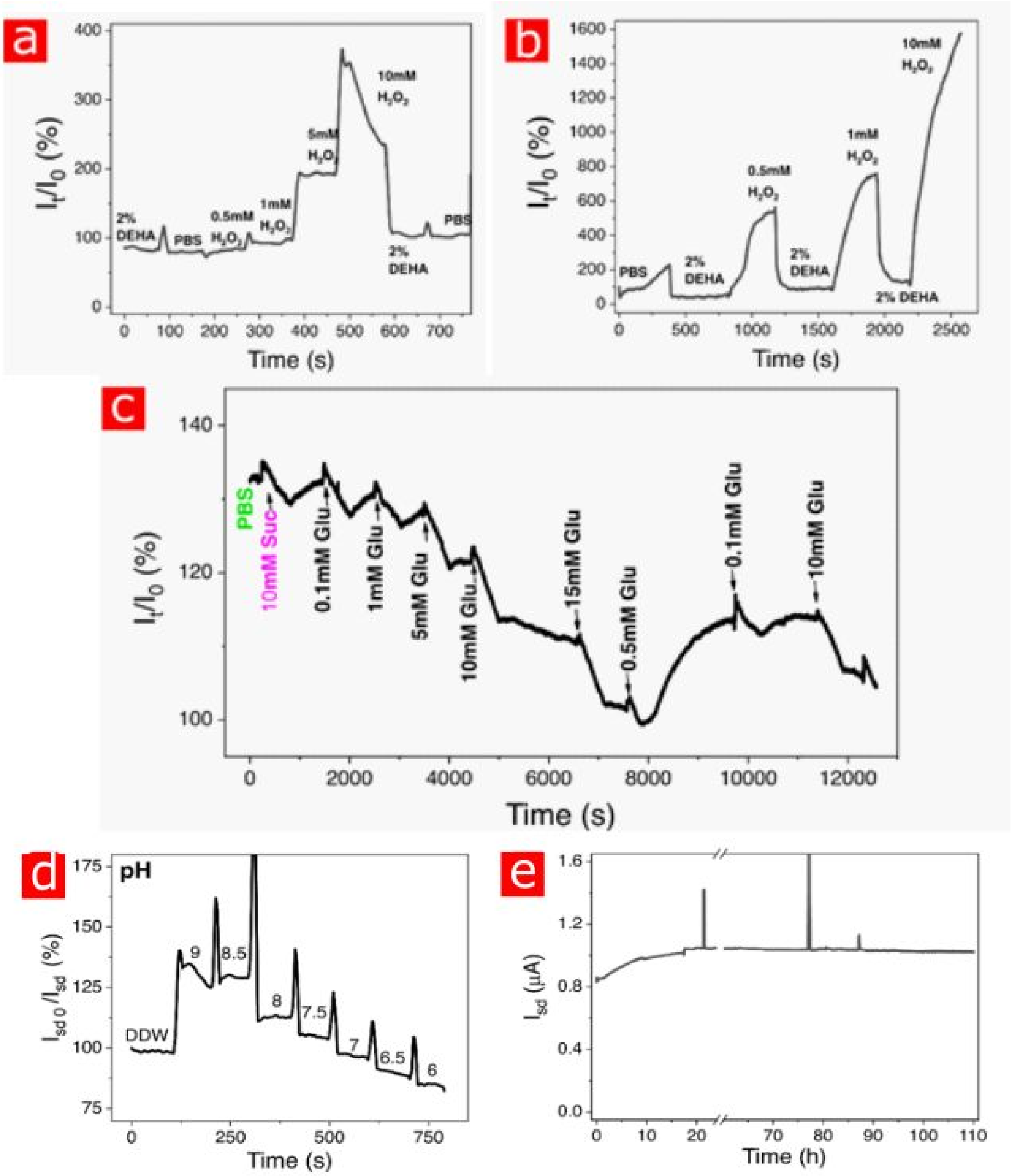

Figure S4| Comparison of SiNW-FET reactivity and stability testing:

a, Microneedle SiNW-based sensors modified with APDMES only. **b**, Microneedle SiNW-based sensors modified with APDMES and AQ. **c**, APDMES-only SiNW-sensor embedded in the hydrogel with GOX. A very small reaction is noticeable due to the lack of sensitivity of APDMES to H_2O_2, as compared to AQ. **d**, pH measurements of the microneedle-based FET after APDMES modification. The pH solution in this experiment was based on 10mM phosphate buffer. **e**, Stability test of SiNW-FET sensor modified with APDMES in PBS solution, for over 4 and a half days**.** The test was continuous for the first 24 hours, was paused for 48 hours, keeping the chip immersed in PBS, and then resuming the electrical measurements for another 48 consecutive hours. The spikes were caused by environmental interferences and are momentary.

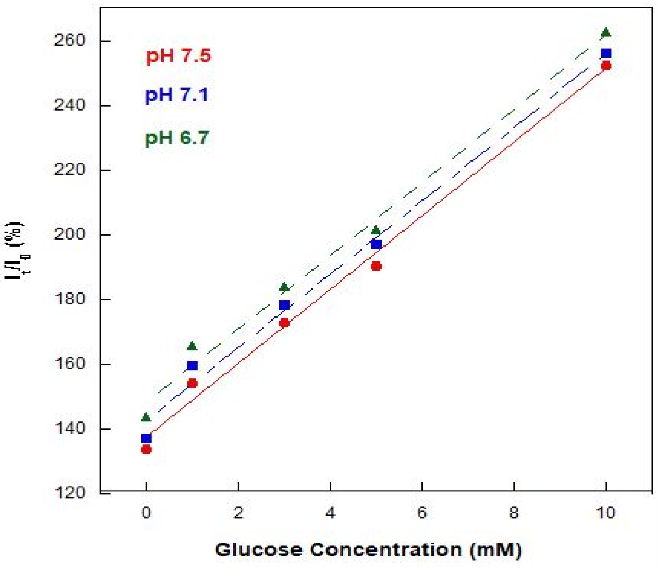

Figure S5 | Microneedle sensor measurement of glucose in hydrogel at different pH:

Reaction calibration measurements for glucose concentrations in hydrogel medium at pH values of 6.7 (green curve), 7.1 (blue curve), and 7.5 (red curve). Measurements were performed with applied gate-reduction, V_g=-0.3V, V_{sd}=0.2V.

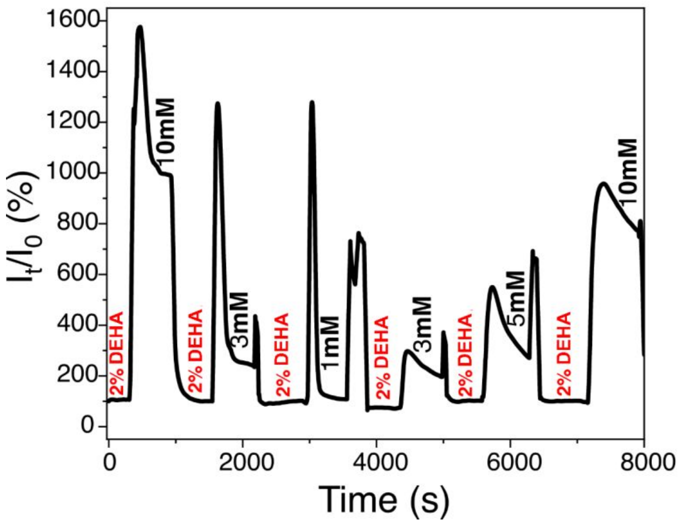

Figure S6 | Microneedle sensor measurement of H_2O_2 in PBS (155mM) containing 10% serum: 3 hour long experiment sensing of 1-10mM H_2O_2. The left part (~0-4000s) display sensing ranging from 10mM to 1mM while the right part rising of the concentration from 1mM to 10mM. Every exchange of H_2O_2 followed by withdraw of the solution and insertion of 2% DEHA in PBS.

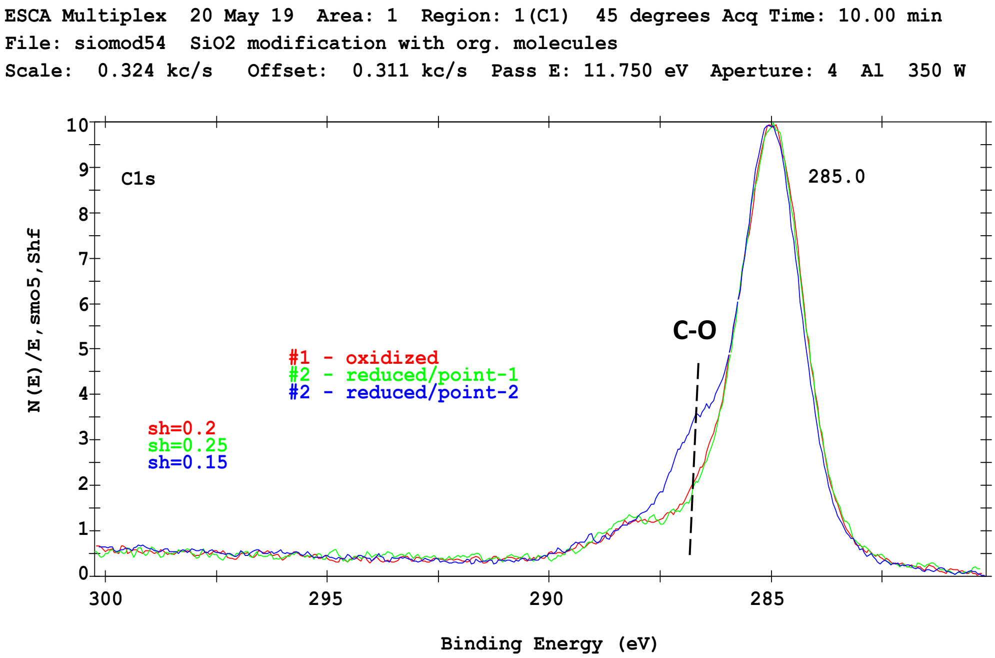

Figure S7 | XPS of AQ molecules reduction by gold electrode:

A silicon substrate with 300nm thermal oxide was modified with AQ by APDMES linker. The substrates were immersed in 10mM of H_2O_2 in PBS solution. Sample #1 and sample #2 were immersed in PBS for 5 minutes, washed by ddw, and dried under N_2. Sample #2 continued for electro-reduction in PBS by grounding the sample on one side and immersing the other in solution with gold electrode. A voltage of -0.6V was applied on the gold electrode for one minutes. Sample #2 was washed again as previously. Sample #1 (red line) was measured in one spot while sample #2 was measured on two spots (blue and green line). The blue plot represents the spot on the side which was inside the solution during electro-reduction while the green plot the spot outside of the solution.

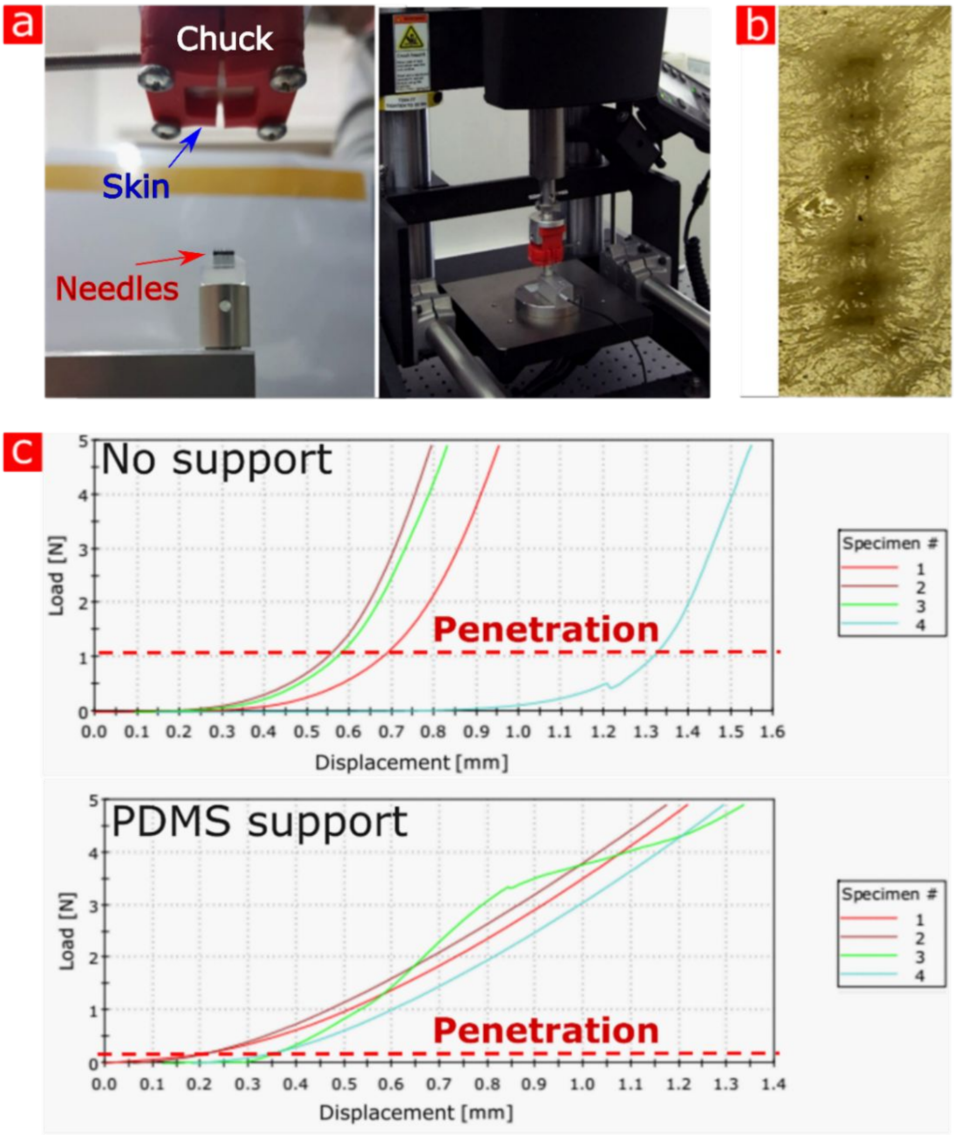

Figure S8 | Microneedles insertion in pigskin:

a, Instron 5948 – Microtester Precision Instrument for small scale testing. The chuck stretches the skin to assimilate the forearm skin, and has an exposed area of the skin, which permits the microneedle chip to travel. **b,** Pigskin penetration pores after insertion and extraction. All microneedles were intact and no residues were visible. **c,** Plots describing forces measured as a function of chip vertical displacement, with each experiment repeated 4 times. In order to estimate forces required for skin penetration, we have performed both free standing skin and PDMS supported skin test, simulating different skin behaviors (the latter is stiffer due to the support).

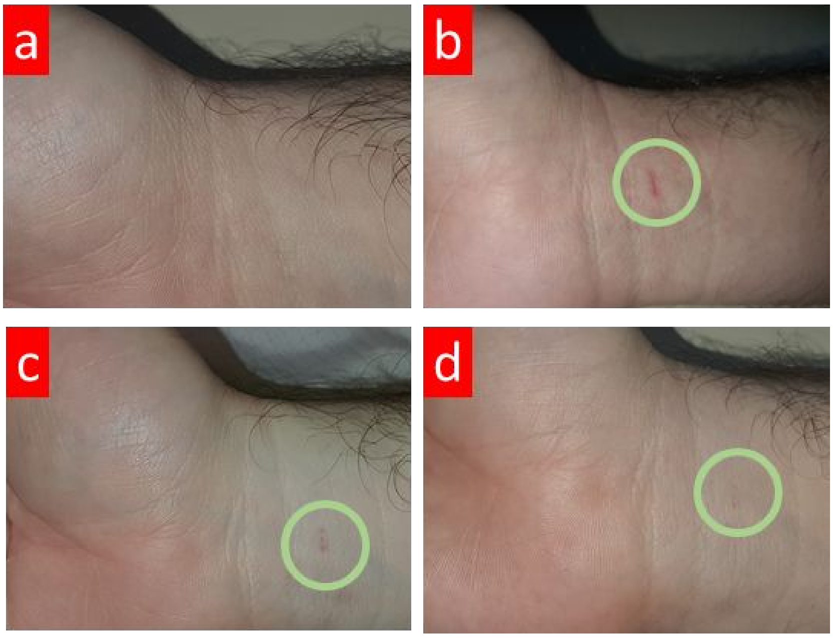

Figure S9 | Skin reaction to insertion

a) Area before insertion. b) Area immediately after extraction of the microneedle. c) Area After 3 hours. d) Area after 24 hours.

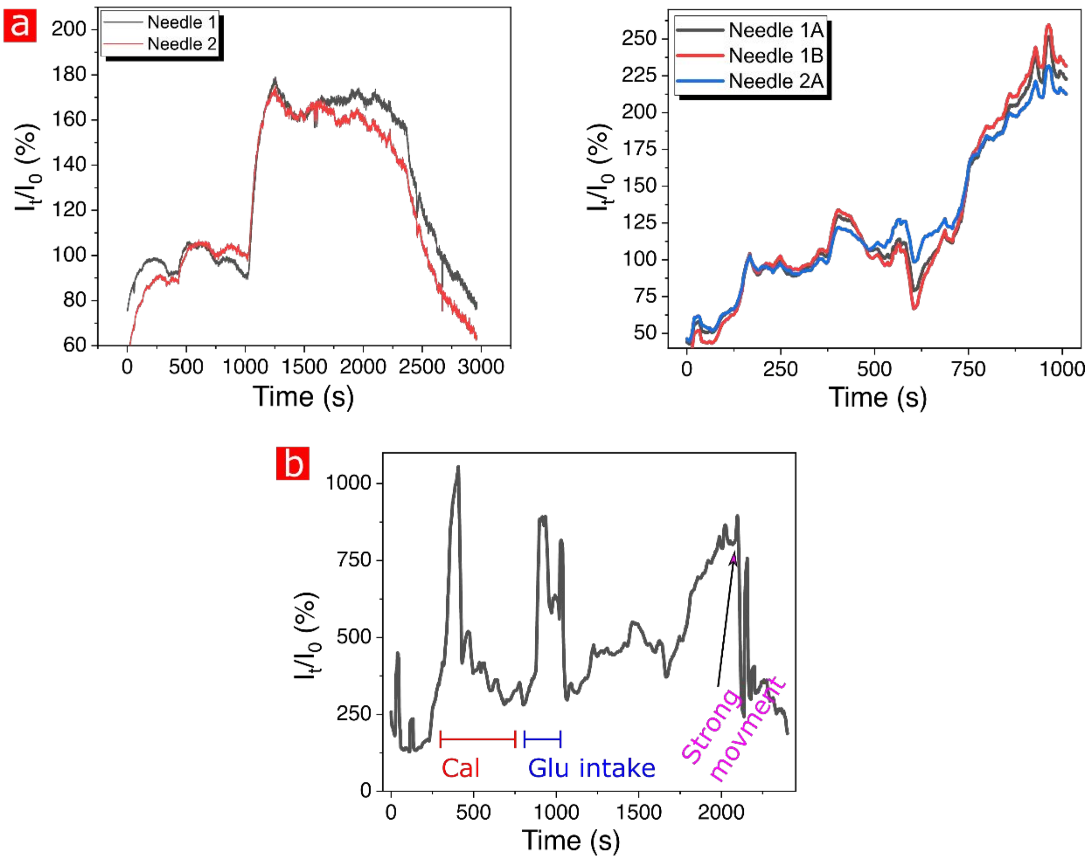

Figure S10 | *In-vivo* CGM additional information:

a, Similarities in reactions of different individual microneedles during *in vivo* experiments, after normalization of the data. The variance in currents is shown to be less than 10% and similar behavior is exhibited in each needle. **b,** changes in currents affected by movements in the subject arm during *in vivo* experiment. These changes are shown to be reversible, as the current quickly returns to its former state.

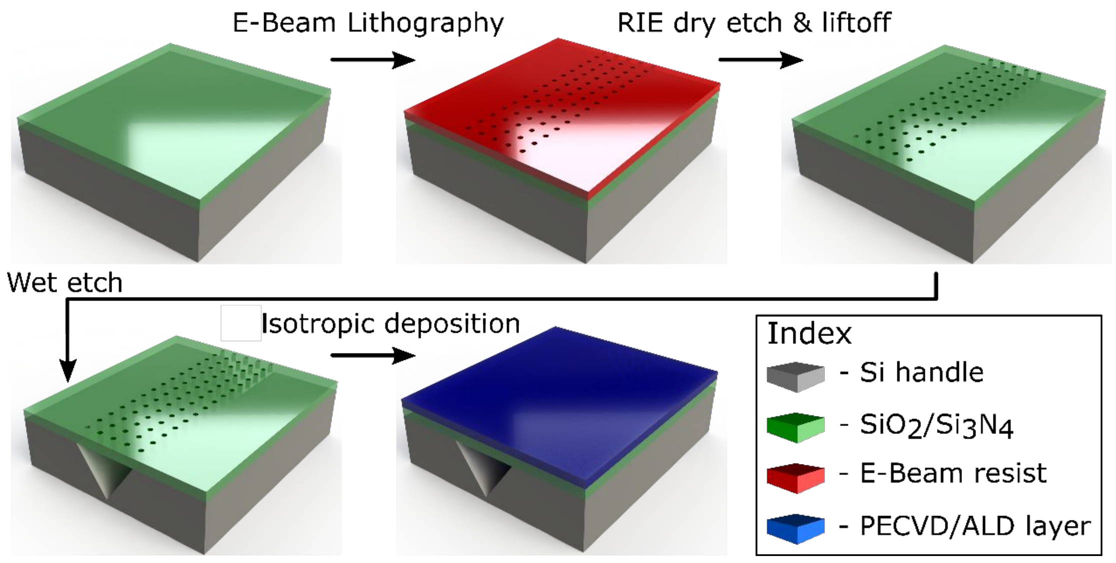

Figure S11 | Microchannels sample fabrication steps.

a) Si substrate with SiO_2/Si_3N_4 top-layer is cleaned; b) EBL process open pores windows; c) RIE etch pores exposing the Si, resist lift off and chip cleaning; d) sample is immersed in 10% TMAH at 65° for one hour to create the roofed microchannel. This step can be performed after needle micromachining; e) Sample is washed and cleaned, followed by isotropic deposition until pores are sealed.
